# Powering the next industrial revolution: transitioning from nonrenewable energy to solar fuels *via* CO_2_ reduction

**DOI:** 10.1039/d0ra07790a

**Published:** 2020-12-22

**Authors:** Rami J. Batrice, John C. Gordon

**Affiliations:** Chemistry Division, Inorganic, Isotope, and Actinide Chemistry, Los Alamos National Laboratory Los Alamos New Mexico 87545 USA batricer@lanl.gov jgordon@lanl.gov

## Abstract

Solar energy has been used for decades for the direct production of electricity in various industries and devices; however, harnessing and storing this energy in the form of chemical bonds has emerged as a promising alternative to fossil fuel combustion. The common feedstocks for producing such solar fuels are carbon dioxide and water, yet only the photoconversion of carbon dioxide presents the opportunity to generate liquid fuels capable of integrating into our existing infrastructure, while simultaneously removing atmospheric greenhouse gas pollution. This review presents recent advances in photochemical solar fuel production technology. Although efforts in this field have created an incredible number of methods to convert carbon dioxide into gaseous and liquid fuels, these can generally be classified under one of four categories based on how incident sunlight is utilised: solar concentration for thermoconversion (Category 1), transformation toward electroconversion (Category 2), natural photosynthesis for bioconversion (Category 3), and artificial photosynthesis for direct photoconversion (Category 4). Select examples of developments within each of these categories is presented, showing the state-of-the-art in the use of carbon dioxide as a suitable feedstock for solar fuel production.

## Abstract 2

Solar energy has been used for decades for the direct production of electricity in various industries and devices. However, harnessing and storing this energy in the form of chemical bonds has emerged as a promising alternative to fossil fuels.

## Graphical abstract

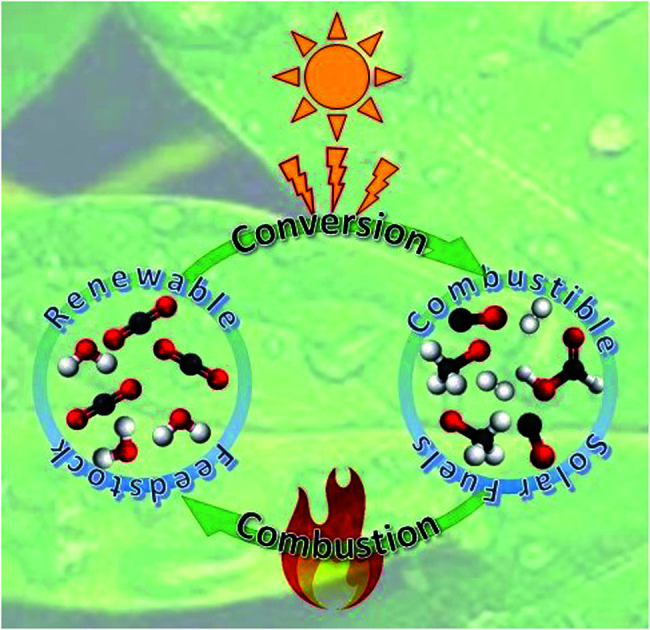

## Introduction

In the more than 300 000 years of human existence,^1^ no time period has been characterised by more rapid growth and technological advancement than the Industrial Revolution which began in the 18^th^ century. This time period up to present day, commonly referred to as the Anthropocene,^2^ has been witness to profound advances in society which has allowed for: (1) greater affordability and accessibility to goods, (2) the development of labour-saving inventions, (3) rapid growth in medical understanding and technologies, (4) greater wealth and improved quality of life for the average person, and (5) increased supply and demand for specialized professions. These advances coupled with high levels of urban migration ultimately provided for an increased global carrying capacity, leading to unprecedented population growth from 791 million prior to the Industrial Revolution (1750) to 7.795 billion in 2020.^3,4^ While the quality of life for some, especially those in the legacy world, has increased dramatically in light of such innovations, several challenges have risen from the nearly ten-fold increase in population, not the least of which is the substantially greater energy demands required to power our modern society.

**Fig. 1:**
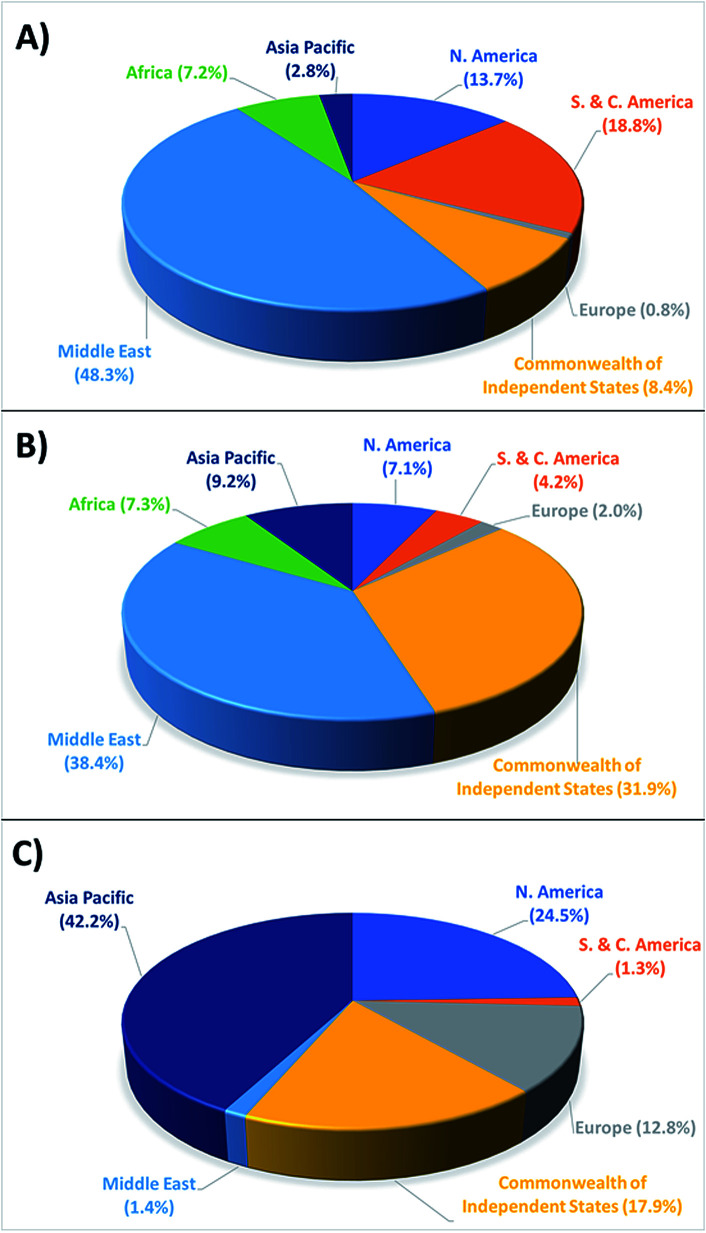
2018 regional distribution of proved (a) oil reserves, (b) natural gas reserves, and (c) coal reserves.

Interestingly, the fuels used today to power transportation and industry vary little from those used nearly 300 years ago insomuch as we continue to rely on non-renewable fossil fuels in the form of coal, oil, and natural gas. This has led to two major problems; first, continued and increased use of fossil fuels has greatly diminished the global supply, and while their scarcity remains a point of debate, it is indisputable that they remain a finite and dwindling resource. Moreover, the geographical distribution of proved fossil fuel reserves (Fig. 1)^5^ raises questions about their long-term availability, especially when considering increased geopolitical tensions and mounting international sanctions. The second, and more substantial problem of this observed population growth and associated energy consumption is the proliferation of greenhouse gases (carbon dioxide (CO_2_), methane (CH_4_), nitrous oxide (N_2_O), *etc.*) in the environment and the resulting global climate change. Of these gases, CO_2_ poses the greatest threat, as it is by far the major greenhouse gas produced from fossil fuel combustion contributing to climate change, and also due to its persistence within the atmosphere.^6,7^ Historical records in fact show atmospheric CO_2_ levels rising from 277 ppm immediately prior to industrialisation in 1750, to 417 ppm by April of 2020, marking a nearly 51% increase.^8,9^ This corresponds to a 1.43 °C increase of the average global surface temperature from 13.42 °C to 14.85 °C within this time period,^10,11^ with the most dire projections showing a potential 4 °C increase beyond preindustrial levels by 2100.^12,13^ Even with the increase in temperature and atmospheric CO_2_ levels already observed, catastrophic effects have resulted including: oceans warming (0.11 °C per decade between 1971–2010), glaciers/ice sheets melting (∼4% recession between 1979–2012), sea level rise of 0.19 meters between 1901 and 2010, 26% increase in ocean acidity from the Industrial Revolution to 2014, increased coastal erosion, unpredictable precipitation patterns leading to regional drought/flooding, major disruption of terrestrial and marine ecosystems, increased frequency and intensity of meteorological natural disasters, compromised food supply and security, and forced migration of native populations.^14^ Presented with such data, it is immediately apparent that now, more than ever, the ability to mitigate these hazards is needed.

**Fig. 2:**
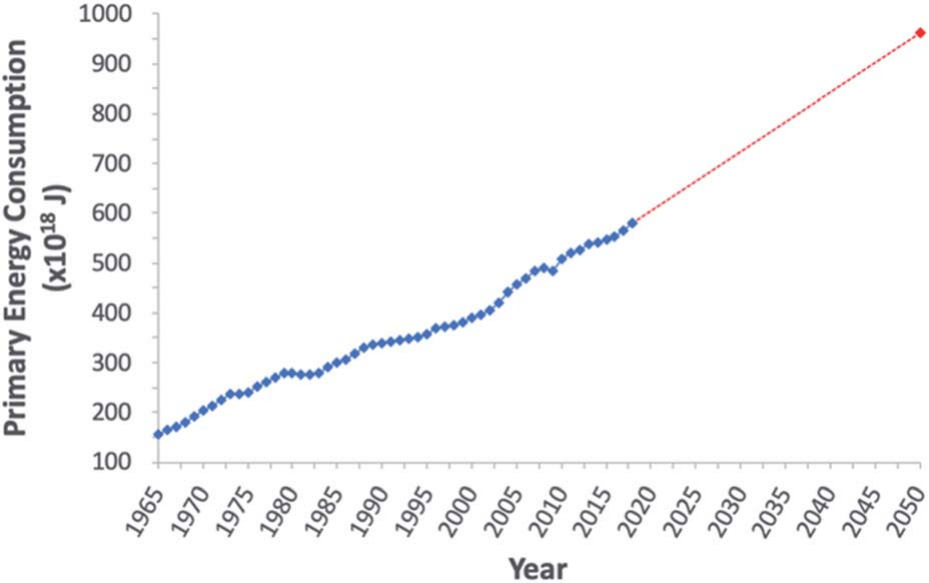
Graphical representation of global primary energy consumption. Blue trend line represents data from 1965–2018. Red dashed trend line shows projected increase between 2018 and 2050.

**Fig. 3:**
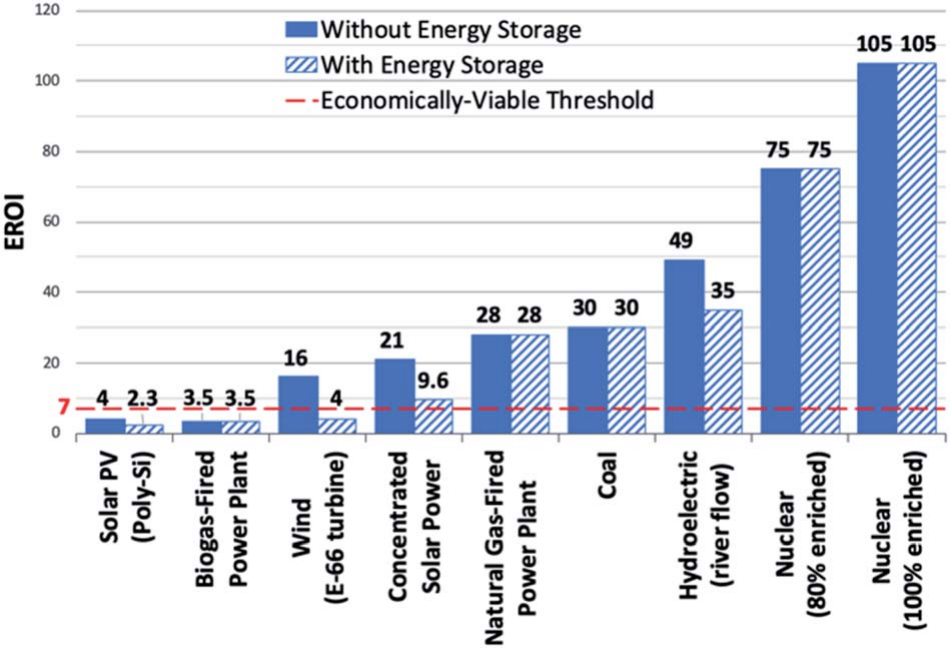
EROI's of electricity generating energy sources. Dashed red line denotes economically-viable threshold. Transportation energy sources not represented in figure.

To address the current climate crisis and growing energy demands (Fig. 2),^15,16^ extensive work has been devoted toward developing renewable energy technologies to either augment or replace fossil fuel use globally. Nonetheless, while the Paris Climate Accords of 2015 showed an apparent willingness of nations to address the growing threats of climate change,^17^ progress since this treaty has been limited – the primary energy consumption generated from renewable sources increased from 9.3% in 2014 to only 10.9% in 2018.^5,18^ In order to meet current demands and prepare for the additional 380.7 exajoules (EJ) needed by 2050, all potential renewable energy sources must be measured against current standards; these sources include wind, hydropower, geothermal, ocean, biomass, and solar power. In determining the portfolio of viable alternative energy sources, three factors must initially be considered: (1) theoretical potential, (2) technical potential, and (3) energy return on investment (EROI). Theoretical potential refers to the upper limits of an energy source available without consideration of any potential restrictions. Technical potential is identified as the usable energy of a given source, limited by factors such as geographical distribution, terrain, land use rights, and others. Finally, EROI is an analytical metric useful for determining the efficiency and viability of an energy producing technology or industry, measured as the ratio of the energy obtained from a resource to the energy expended to produce that energy. Given that EROI is based upon current technologies, it is not necessarily a good indicator for the long-term potential viability of a technology, as future advancements can further increase EROI values. Analysis of these three factors has been studied extensively and given rise to theoretical and technical potentials for all major classes of renewable fuels (Table 1),^19,20^ as well as EROI models which reflect current technologies and economic analyses (Fig. 3).^21,22^ Even limited by the technical potentials, it is clear that solar energy is by far the most abundant and desirable renewable energy source,^23^ providing more than 60 times the 2050 projected annual global energy consumption.

**Table 1:** Overview of theoretical and technical potentials of renewable energy sources in EJ per year

Renewable energy source	Theoretical potential^24^ (EJ per year)	Technical potential^20^ (EJ per year)
Wind	6000	1250–2250
Hydropower	147	50–60
Geothermal	1400	810–1545
Ocean	7400	3240–105 000
Biomass	1548	160–270
Solar	*3* *900* *000*	*62 000–280* *000*
**Total**	**3** **916** **495**	**76** **000–294** **500**

Towards mitigation of the more pressing issue of growing CO_2_ emissions, rising surface temperatures, and the ongoing detriment to the ecosystem caused by these factors, it is most prudent for efforts to be directed at the major source of greenhouse gases. According to 2017 energy statistics from the International Energy Agency, nearly 91% of CO_2_ emissions in this year were due to some form of fuel combustion across all industrial sectors.^25^ Unfortunately, the renewable energies discussed thus far address needs for electricity generation, though they remain invaluable as alternatives for power production in, for example, industrial and residential sectors. However, much of our global economy and livelihood relies heavily on both agriculture and transportation, economic sectors which require liquid fuels rather than direct electricity supply. The most promising emerging technology capable of filling this niche is solar fuels, serving as both a renewable energy source and combustible fuel with net-zero CO_2_ emissions. To date, the only industrial-scale renewable fuels in use are agro-biofuels, further illustrating the potential growth opportunities open in this field of research and industry.

## Solar fuel processes and subsystems

As early as the turn of the 19^th^ century, scientists of the day began uncovering the secrets of photosynthesis and the metabolic pathway by which autotrophs turn water, CO_2_, and sunlight into energy and biomass. Though being able to mimic these ubiquitous processes in a laboratory setting has been little more than a curiosity, recent years have witnessed a massive push to realize this objective,††A database search of the term “solar fuels” showed no more than six published works per year from 1977–2008. Beginning in 2009, the average number of publications per year was more than 134. with some of the earliest reports being published in the late 1970's.^26,27^ While nature's goal is generally to create glucose for plant growth, flower formation, and fruit development, our goal within the scientific community is rather to create solar fuels derived from these simple building blocks.

Current research has led to the generation of solar fuels through two general pathways; either by splitting water to form hydrogen (H_2_) and oxygen (O_2_) gas, or by reducing CO_2_ in the presence of a sacrificial proton donor to generate carbon-based gas or liquid fuels. A cursory examination of the physical properties of some of the most common renewable fuels may point to H_2_ as an interesting option owing to its high energy density and the water which is formed as the sole product of combustion (Table 2); however, a major drawback globally is the lack of existing H_2_ based infrastructure and storage capacity, a shortcoming which would require major engineering overhauls worldwide. Moreover, observing the volumetric energy density of H_2_ compared to renewable liquid fuels, such as methanol, reveals that the H_2_ energy density per litre at STP is in fact 0.013 MJ, whereas the same volume of methanol would release 18.17 MJ upon combustion. The increased energy density of the fuels produced from CO_2_ in addition to the variety of liquid fuels which could be conveniently integrated to our existing infrastructure, point to the use of CO_2_ as the ideal feedstock for solar fuel production. An added advantage to this approach is the product diversity accessible, including gaseous fuels which can be used directly or converted to more useful products using known industrial methods (*i.e.* Fischer–Tropsch fuels), as well as other valuable liquid energy carriers. An initial concern is based upon the fact that creating and using these carbon derived energy sources would result in additional release of CO_2_ upon combustion, however, such an assumption fails to consider that the model for implementation of these technologies necessitates consumption of CO_2_ as a feedstock, making these carbon–neutral fuels (*i.e.* generated CO_2_ is ultimately recycled to create more solar fuels).^32^

**Table 2:** Properties of some common renewable fuels. Hydrogen, methane, methanol, and ethanol are pure compounds. All other fuels are mixtures and values provided represent ranges found in literature^28–30^

Fuel	Phase at STP	Composition	Mass density (kg L^−1^)	Energy density (HHV)^a^ (MJ kg^−1^)
Hydrogen	Gas	H_2_	9.0 × 10^−5^	142
Methane	Gas	CH_4_	6.6 × 10^−4^	55.6
Biogas	Gas	CH_4_, CO_2_, *etc.*	>6.6 × 10^−4^	<55.5
Syngas	Gas	H_2_, CO, CH_4_, CO, *etc.*	9.5 × 10^−4^	3–15^b^
Methanol	Liquid	CH_3_OH	0.79	23.0
Ethanol	Liquid	C_2_H_5_OH	0.79	29.6
Biodiesel	Liquid	Long-chain, mono-alkyl esters	0.88	37.5
Traditional biomass fuel	Solid	Woods, charcoal, agricultural residues, dung, ash	0.6	18
Dry green microalgal biomass	Solid	Proteins, carbohydrates, lipids, ash	0.6	20

a Higher heating value: energy released as heat given complete combustion at STP.

b HHV is highly variable due to different ratios of component gases and technology used to produce mixture.^31^

**Fig. 4:**
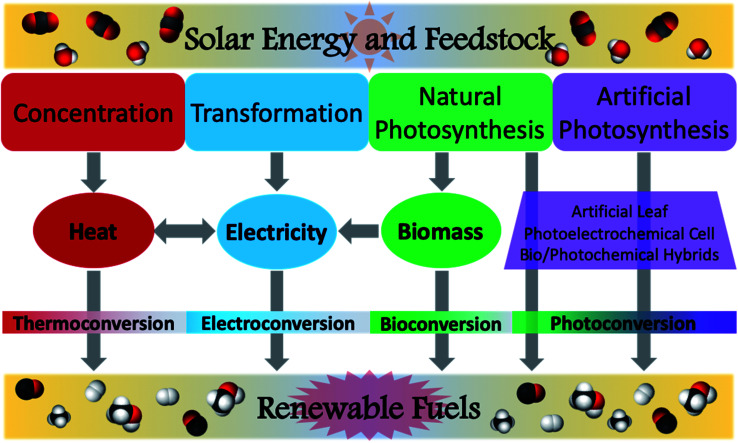
Modes of solar energy conversion of carbon dioxide and water into renewable fuels. Concentration (Category 1): solar radiation is concentrated for the generation of heat; generated heat can be used for thermochemical conversions or toward the generation of electricity. Transformation (Category 2): harnessed solar energy is converted into electricity, then used to power an electrochemical cell. Natural photosynthesis (Category 3): autotrophic conversion of H_2_O and CO_2_ into biomass, or direct conversion of feedstock into viable fuels. Artificial photosynthesis (Category 4): use of photoactive materials to harvest sunlight and achieve photoconversion of carbon dioxide and water to renewable fuels. Figure contents and layout adapted from previous literature.^39^

Despite this field of study having started in earnest merely a decade ago, extensive research has produced impressive results toward realizing this goal and mitigating the growing climate crisis.^23,33–36^ In assessing the feasible use of various solar fuel technologies, several studies have been conducted to determine factors such as system efficiencies or shipping/transportation costs.^37,38^ However, this review will focus on recent advances in the photochemical conversion of CO_2_ using natural or simulated solar radiation as the light source. In characterising the reactions to achieve this transformation, it is useful to identify the disparate activation pathways which facilitate the conversion of the feedstock to target chemicals. This was aptly done in a recent review by van der Zwaan and co-workers, wherein a taxonomical classification was developed for the characterisation of solar fuel generation according to the mode of solar energy harvesting.^39^ This was conveniently identified as Categories 1–4 (Fig. 4), where Category 1 is identified as concentration, Category 2 as transformation, Category 3 as natural photosynthesis, and Category 4 as artificial photosynthesis. Solar concentration (Cat1) relies upon harvesting solar energy and focusing the incident radiation to generate heat. While the most basic version of this can be demonstrated using a magnifying glass on a sunny day, modern solar concentrators are remarkably sophisticated and continue to be refined. In a further embodiment, transformation (Cat2) is characterised by the conversion of solar radiation into an electrical current; though this category is rather ubiquitous in the form of photovoltaic cells, in the context of solar fuels, this describes the generation of a voltage potential to power an electrochemical cell for the production of solar fuels. Category 3 encompasses natural photosynthetic processes by plants, cyanobacteria, algae, and other autotrophic organisms. This often produces glucose as the main carbon fixation product, useful for biomass generation, but biofuels may also directly or indirectly be generated from many such organisms. Modern advances in biotechnology have allowed for the creation of genetically modified and bioengineered organisms suitable for selective production of solar derived biofuels. While these transgenic species are not naturally occurring, their use for solar fuel production is typically considered to fall under the rubric of natural photosynthesis. Discussions of Cat3 within this review will therefore follow this convention and consider both wild-type and mutant species, as well as genetically modified organisms (GMO's). Artificial photosynthesis (Cat4) is inspired by the processes observed in Cat3, but has been tailored to harvest light energy and directly generate the desired solar fuels using either laboratory-prepared materials or bio-synthetic hybrids. Despite each of these categories being distinctly identified, several solar fuel systems have been developed which encompass more than one of these categories; these will not be discussed in detail in order to succinctly elaborate upon advances within each of the individual components.

In examining the pathways to fuel generation from each of these solar conversion methods, water splitting toward H_2_ generation has arguably received the most attention and produced several impressive works.^40–52^ Nonetheless, as aforementioned, CO_2_ fixation and conversion better addresses current environmental needs while easily integrating into current infrastructures. Thus, the fuels of focus will generally be those presented in Table 2, as well as formate/formic acid. While the latter is not typically considered a fuel source directly, formate has been found to power novel designs of fuel cells,^53–58^ as well existing as an intermediate or byproduct of the catalytic conversion of CO_2_ to methanol (MeOH).^59–62^ Though the photochemical conversion of CO_2_ may be achieved by numerous synthetic routes, most work has been devoted to the hydrogenation of this substrate to oxygenates and/or hydrocarbons by pathways resembling a Sabatier (eqn (1)) or reverse water–gas shift reaction (RWGS) (eqn (2))

1 CO_2_ + 4H_2_ → CH_4_ + 2H_2_O

2 CO_2_ + H_2_ → CO + H_2_O

Alternatively, products of photochemical reactions may be further converted to higher value fuels by methods such as the Fischer–Tropsch (FT) conversion of syngas into pure hydrocarbons.

### Category 1: thermoconversion by solar concentration

The warming effect of sunlight is a concept which is easily understood by all manner of species across the globe. As such, it is only logical that solar radiation can be harnessed to generate the thermal energy necessary to overcome enthalpic barriers for the chemical conversion of CO_2_ into combustible energy carriers. Currently, this may be achieved *via* one of two general pathways – either through indirect or direct thermoconversion, with several examples of each appearing regularly in scientific literature.^63–68^ Direct thermoconversion makes use of photonic thermomaterials which readily absorb incident light and convert this energy to heat, creating a localised temperature gradient at the site of reactivity. Conversely, indirect thermoconversion employs the use of solar concentrators which focus incident light energy (and where desired, filter the radiation to utilise a more narrow wavelength range) to a more concise area in order to heat the bulk reaction media. Developments in methods of direct photothermal reactions have relied heavily on research in pure and applied chemistry, whereas indirect photothermal reactions have advanced as a result of improvements in the engineering and design of solar concentrators. This section will focus more broadly on recent advances in direct thermoconversion, however developments in indirect thermoconversion processes will be discussed.

#### Direct thermoconversion: photonic materials

In a recent report by Wang *et al.*, cupric sulphide doped titania was prepared by the hydrothermal reaction of CuCl_2_·2H_2_O, thiourea, and anatase TiO_2_.^69^ The resulting nanosheet product was identified as a 2% doped material, which was then deposited as a water suspension on glass slides and exposed to an atmosphere of CO_2_ under UV-Vis-NIR irradiation. Pure titania nanosheets were found to produce CO as the main product at a reaction rate of 3.39 μmol g^−1^ h^−1^, whereas the 2% CuS doped material drastically increased reactivity to 25.97 μmol g^−1^ h^−1^. Temperature measurements obtained at the catalyst surface revealed that light irradiation resulted in a temperature increase up to 138 °C, and cooling the temperature of the reaction resulted in notable decreases in reaction rates, supporting the photothermal characteristics of the chemical conversion. Fourier-transform infrared spectroscopy of the product mixture also showed the formation of monodentate carbonate (m-CO_3_^2−^), polydentate carbonate (p-CO_3_^2−^), carboxylates including bicarbonate and formate, and formic acid as minor byproducts. While in this study, a standard glass slide was used as the substrate, another investigation by Ozin and co-workers observed the enhanced reactivity of hydroxylated indium oxide deposited on silicon nanowires (In_2_O_3−*x*_(OH)_*y*_/SiNW) when compared to similar catalysts on roughened glass substrates.^70^ Use of a vertically aligned silicon nanowire support minimised the reflection losses and enhanced light trapping, resulting in photothermal heat generation up to ∼160 °C within a few minutes, and a nearly 6-fold increase in the CO production rate up to 22.0 μmol g^−1^ h^−1^. The temperature dependence on the preparation of the photocatalyst was studied by Zhao *et al.* in the photothermal generation of CO by a FeO–CeO_2_ nanocatalyst.^71^ Fe(OH)_3_–Ce(OH)_3_ precursors were reduced using H_2_ in the range of 200–600 °C, yielding the nanocomposite catalysts. The material prepared with a 2 : 1 ratio of Fe : Ce at 300 °C (Fe_2_Ce1-300) showed the greatest activity, with a CO_2_ conversion of 43.63%, CO selectivity of 99.98%, and a reaction rate of 19.61 μmol g^−1^ h^−1^. When the reduction temperature is increased in the range of 300–500 °C, the reaction rate and product selectivity both decrease, generating greater amounts of CH_4_ owing to the formation of Fe^0^ during the catalyst synthesis.

**Fig. 5:**
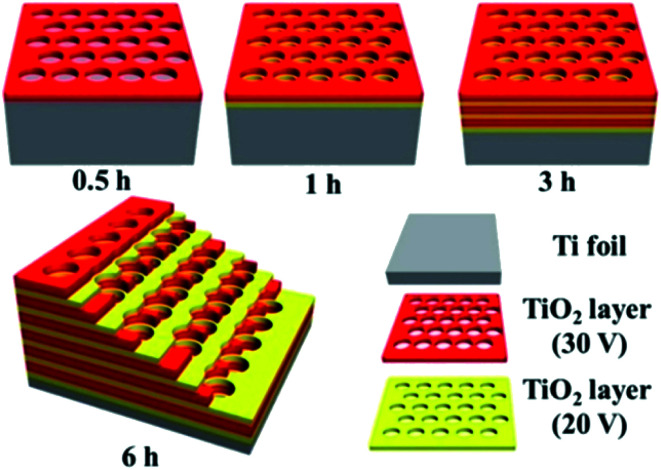
Schematic diagram of titania photonic crystals prepared by anodisation and calcination of titanium foil. Image adapted with permission from ACS Publications.^72^

In order to optimise the amount of CH_4_ produced instead of carbon monoxide, other light-absorbing catalysts and reaction systems have also been investigated to these ends. Research from the Yu group sought to identify a phase of titania ideal for achieving thermal photoconversion of CO_2_ into viable solar fuels; using methods developed in their work, titania photonic crystals were formed by anodisation of titanium foil with alternating voltages of 20 and 30 V in thirty minute increments, followed by annealing at 550 °C for two hours, yielding macroporous photonic crystals with air cylinder diameters of 100 nm (Fig. 5).^72^ Systematic studies were performed using various phases of TiO_2_, revealing that the anatase titania photonic crystals prepared in this study significantly outperformed both P25 and titania nanotube arrays in the photomethanation of CO_2_ (35.0, 2.2, and 7.5 μmol h^−1^ m^−2^, respectively). The improved reactivity was attributed largely to the slow photon effect, wherein the incident light energy is slowed and propagated more efficiently due to interactions between the light and medium,^73^ in this case the photonic crystals. Improving even further upon this observed activity, Ye and co-workers presented the use of solid supports doped with various group VIII metals. Impregnation of ultrathin Mg–Al layered double hydroxides with ruthenium nanoparticles generated the highly stable catalyst Ru@FL-LDH.^74^ Introduction of this catalyst into a flow photoreactor in the presence of CO_2_, H_2_, and light irradiation resulted in photothermal methanation at rates as high as 277 mmol h^−1^ g^−1^, with sustained CO_2_ conversions of approximately 95%, and nearly 100% selectivity for CH_4_ production. Under sustained irradiation, Ru@FL-LDH maintained a surface temperature of roughly 350 °C and impressively retained its catalytic activity over several cycles of substrate introduction. Using an even more ubiquitous support, mesoporous Al_2_O_3_ was doped with numerous transition metal nanoparticles including Ru, Rh, Ni, Co, Pd, Pt, Ir, and Fe.^75^ The catalysts produced (M/Al_2_O_3_) were similarly placed in an atmosphere of CO_2_ and H_2_ under light irradiation, resulting in rapid photon-mediated heating of the catalyst nanoparticles (300–400 °C) and highly selective photomethanation of CO_2_. The most notable results were found with the alumina-supported ruthenium, rhodium, and cobalt nanoparticles, revealing a maximum CO_2_ reaction rate of 18.16 mol h^−1^ g^−1^ using Ru/Al_2_O_3_, CO_2_ conversion of 96.25% with Rh/Al_2_O_3_, and a CH_4_ selectivity of 99.51% when the reaction was performed with Co/Al_2_O_3_. Interestingly, it was found that these three catalysts in addition to the nickel analogue (Ni/Al_2_O_3_) significantly outperformed the palladium, platinum, and iridium nanocatalysts.

#### Indirect thermoconversion: concentrated solar power

**Fig. 6:**
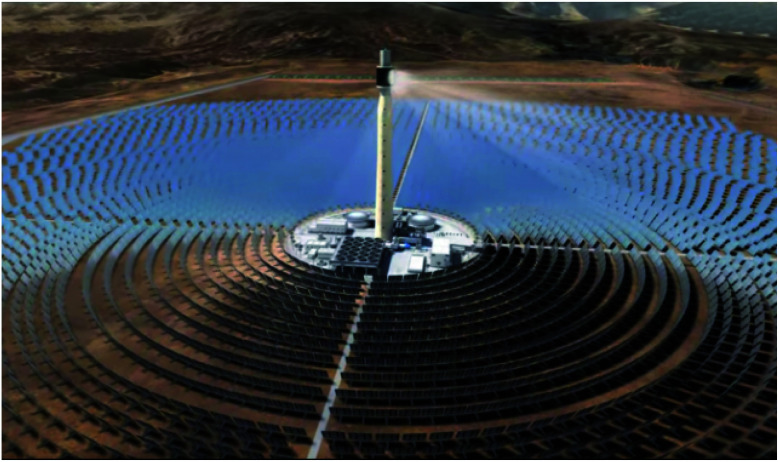
Aerial view of the Quarzazate Solar Power Station in Morocco. Image adapted from cited ref. 76.

**Fig. 7:**
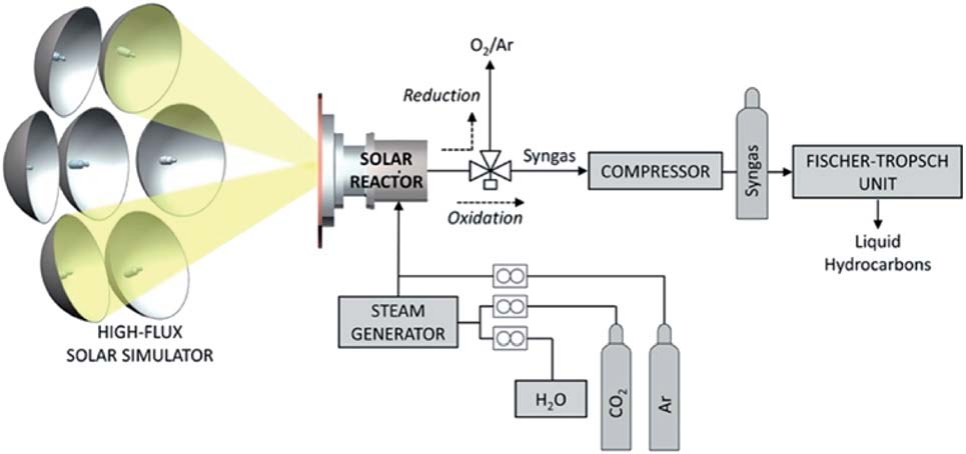
Schematic diagram of solar reactor used in the ceria-mediated photothermal reduction of carbon dioxide. Image adapted with permission from ACS Publications.^79^

**Fig. 8:**
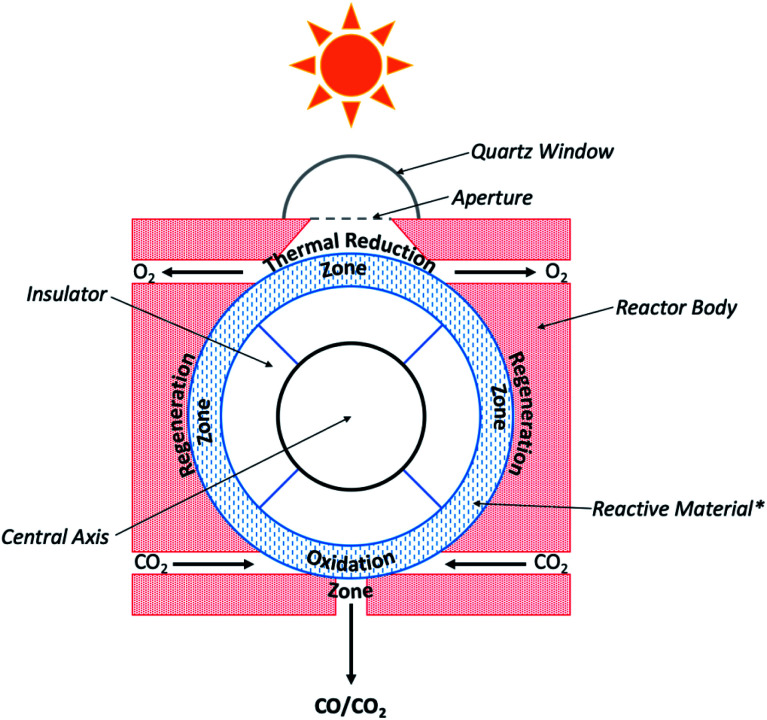
Schematic diagram of CR5 used for CO_2_ photoconversion. *Reactive material is comprised of sets of counter-rotating rings. Current cross-sectional view shows single ring oriented coincident with the plane of this page.^83,84^

In contrast to direct thermoconversion by photonic materials, a field of study which largely resides within the realm of research and development, indirect conversion is already an industrialised process which is used broadly across the globe. More colloquially, such photoconverters are known as concentrated solar power plants (CSP), the largest being the Ouarzazate Solar Power Station in Morocco which occupies 2500 hectares (Fig. 6),^76^ with even larger CSPs either in planning or construction phases in Australia and the United Arab Emirates. These CSPs are dedicated to power production and often produce hundreds of megawatts of energy,^77^ however, the same scientific theory which powers these structures may similarly be applied to the production of solar fuels. In the lab-scale systems which investigate these reactions, enclosed reactors are often employed which generally make use of lenses to concentrate light energy rather than the large heliostats used in CSPs; a similar system of parabolic and/or rotating mirrors could also be incorporated to such reactors in order to optimise CO_2_ reduction, though the small reaction sizes used in experimental samples often renders use of a heliostat impractical for such scales. A recent example of a photothermal reactor constructed for the purpose of solar fuel production and incorporating glass lens concentrators was constructed by Han *et al.*^78^ The system studied observed the activity of either titania or titania supported platinum (1% loading) as a function of the catalyst distance from a focusing lens. The lens used was constructed of PMMA and permitted 92% light transmittance, and the photocatalytic conversion of CO_2_ into CH_4_ was measured at a catalyst-to-lens distance (DLC) of 5, 10, 15, 20, and 25 mm. For the constructed system, the researchers determined that the greatest activity was observed at DLC15 for both TiO_2_ and Pt/TiO_2_ with CH_4_ yields of 18.12 and 20.55 μmol g^−1^, respectively. Additionally, the quantum efficiencies (*Φ*) for the catalytic Sabatier reactions were calculated according to the following relationship (eqn (3)):3
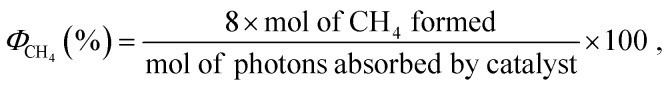
and found to be 8.85 and 10.03%, respectively. In an alternative design, Marxer and co-workers utilised a previously developed solar reactor consisting of a cavity receiver loaded with a ceria reticulated porous ceramic (RPC), and a compound parabolic concentrator (Fig. 7).^79^ The morphology of the ceria used in this study was characterised as a dual-scale reticulated porous ceramic (DS-RPC), wherein the ceramic contained both small-scale (∼10 μm) and large-scale pores (∼0.5 cm); the solar concentrator used in this system was used to boost the incident light intensity to mean values up to 3000 suns through the aperture of the reactor. This design generated a nominal cavity temperature of 1547 °C, and when a feedstock of CO_2_ and H_2_O vapor was introduced to the reactor, resulted in peak CO generation rates of 45 mL h^−1^ g^−1^, nearly double the rate previously reported (22.2 mL h^−1^ g^−1^) from single-scale reticulated porous ceramics (SS-RPC).^80^ Marxer *et al.* further demonstrated the utility of the DS-RPC solar reactor design by integration with a gas compressor and a Fischer–Tropsch reactor system. The syngas generated over the course of 243 reaction cycles was compressed to a pressure of 15 MPa, corresponding to 700 litres of syngas at STP of the following composition: 33.7% H_2_, 19.2% CO, 30.5% CO_2_, 0.06% O_2_, 0.09% CH_4_, and 16.5% Ar. Excess H_2_O was removed from this mixture by condensation, and analysis of the H_2_/CO molar ratio revealed a value of 1.76, a suitable syngas composition for subsequent FT-synthesis. The crude gas mixture was processed in a FT reactor using a cobalt catalyst and yielded 40.6 g of liquids and 48.1 g of solid waxes. Hydrocracking of the waxes ultimately yielded 17.2 wt% naphtha, 35.6 wt% kerosene, 17.1% red diesel, and 30.2 wt% of high boiling heavier fractions (>370 °C). This extensive study represents the first example of jet fuel production by thermoconversion of CO_2_ and H_2_O. Other works from the Steinfeld lab, where this work was performed, has also probed further into the syngas production using this reactor system and shown that; (1) stable and rapid fuel generation has been demonstrated over 500 reaction cycles with little to no loss of ceria reactivity; (2) the solar-to-fuel efficiencies (STF_total_) ranged from about 0.7–0.8% and were found to be limited by system scale and design; (3) the H_2_/CO syngas ratios are tunable in the range of 0.25 to 2.34 by adjusting the H_2_O/CO_2_ feedstock ratio from 0.8 up to 7.7.^81,82^ Using similar theories, work by the Maravelias research group as part of the *Sunshine to Petrol* (*S2P*) initiative has developed a photothermal process for the thermal conversion of CO_2_ to solar fuels, namely methanol and FT fuels derived from syngas feedstocks.^83,84^ At the core of this process is the CR5 solar chemical heat engine (Fig. 8) which operates under the principles of metal-mediated redox conversion of CO_2_ at elevated temperatures. An analysis of the economic viability of the system presented detailed parameters for the thermoconversion by the CR5 and subsequent chemical conversion of the syngas product. This device consists of 102 counter-rotating rings comprised of a judiciously selected reactive material; each ring measures 36 inches (91.4 cm) in diameter, and the ring stack is further enclosed within the reactor body. Previous work by Nakamura identified an ultra-high temperature iron oxide cycle capable of splitting water,^85^ however this redox system was also found to be suitable for the reduction of CO_2_. Using FeO/Fe_3_O_4_, the steps of the catalytic cycle for this reaction follow eqn (4) as described:

4a Fe_3_O_4_ → 3FeO + ½O_2_

4b 3FeO + CO_2_ → Fe_3_O_4_ + CO

4c CO_2_ → CO + ½O_2_.

**Fig. 9:**
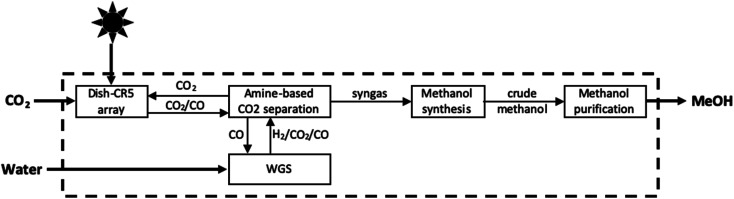
Block flow diagram of photothermal conversion of CO_2_ to methanol by *S2P* process.^84^

In this reaction, eqn (4a) is the high temperature thermal reduction of the iron catalyst, eqn (4b) is the comparatively lower temperature oxidation of ferrous oxide by CO_2_, and eqn (4c) represents the net CO_2_ splitting reaction. In order to generate the high temperatures needed to achieve the initial reduction step, the CR5 is integrated with an 88 m^2^ parabolic dish, giving rise to the Dish-CR5 system. The CO produced from the photothermal process described above is further processed in the subsequent subsystems which include: (1) a WGS reaction loop, (2) an amine-based CO_2_ absorption/separation system, (3) a methanol synthesis reactor, and (4) the methanol purification system (Fig. 9). The WGS reactor uses the CO produced from the Dish-CR5 in combination with a water feedstock to produce a H_2_/CO_2_/CO syngas mixture. Bubbling the gas mixture through aqueous monoethanolamine removes the CO_2_, a well-established industrial practice for removing CO_2_ in coal-fired power plants. The purified syngas mixture is reacted over a Cu/ZnO/Al_2_O_3_ catalyst in a fixed bed reactor to produce crude methanol, followed by purification of the methanol through a flash vessel to remove gaseous byproducts and starting material, then standard distillation to achieve the methanol–water separation, yielding 99% pure methanol. Using this setup, a 1050 hectare methanol production facility is proposed which would include a 17 622 array of Dish-CR5 reactors including the four subsystems necessary to convert the syngas to methanol. The primary solar energy efficiency of this process was calculated to be 7.1%, significantly higher than other comparable thermoconverters, and the production capacity of this facility is expected to be 82 700 Mg of methanol per year, the energetic equivalent of about 51.2 million litres of gasoline (13.5 million US gallons).

### Category 2: electroconversion by solar transformation

Electroconversion is a ubiquitous process owing to the widespread use of commercial and residential solar panels as well as other photovoltaic cells. Rather than using electroconversion toward large scale electricity production, however, the generated voltage may also be utilised downstream in the form of solar fuels. This approach thus combines the broader fields of photo- and electrochemistry and makes use of photoelectrochemical cells to store the electrical energy produced in the form of chemical bonds in combustible fuels.^86,87^ As with many solar fuel technologies, the splitting of water for the generation of dihydrogen has generally received the most attention and produced several notable works.^40,42,44,45,49,88–90^ Unlike the water splitting reaction achieved by PEC systems, CO_2_ reduction is considerably more complex and presents challenges not otherwise present when H_2_O alone is the substrate of interest.^91–101^ First, the generally low solubility and mass transfer of CO_2_ in aqueous and organic electrolytes limits the productivity of most PEC cells. Additionally, when performing the reduction in the liquid phase, only C1 products are detected.^102^ While we have seen that even C1 products of these reactions can serve as solar fuels or easily be converted into suitable energy carriers, decades of research have revealed that performing this class of reaction at the gas–solid interface (gas-phase reaction medium) is capable of generating ≥C2 products. The steps leading to this realisation have required discovery and extensive optimisation to determine the components necessary to create an efficient PEC cell, and current research continues to try and improve upon this technology. This has (generally) resulted in either sophisticated systems which incorporate light harvesting materials into electrode designs (integrated PEC cell), or wiring a photovoltaic cell to an electrolyser which performs the CO_2_ reduction reaction (modular PEC cell). Regardless of which approach is used, the constituents of a PEC cell at its most basic level include a light harvester, electrodes, and a catalyst, though each of these is not necessarily mutually exclusive and depends on the cell design. Within the construction of each PEC cell, these components operate in a highly synergistic manner wherein slight variations may result in drastic differences in properties such as efficiency, under/overpotential required, product distribution, and more. As such, the development of PEC cells for solar fuel generation has produced a diversity of works which have arduously investigated unique photoelectrolyser components and designs.

#### Photoelectrochemical syngas production

Bai and co-workers have investigated the efficacy of various bismuth oxyhalides as photocatalyst for CO_2_ reduction and as photodegradation catalysts for model organic pollutants, most notable of these being the bismuth oxyiodides.^103–105^ This class of compounds proved capable of mediating the production of CO from CO_2_, however the conversions remained modest at best. It was evident from initial studies that BiOI in particular, while displaying impressive light harvesting properties, was ineffective at generating the necessary electron–hole pair to efficiently achieve electrochemical reduction of CO_2_ to CO in aqueous media (*R*_CO_ = 5.18 μmol g^−1^ h^−1^). In another study to follow, BiOI was used as a chromophoric scaffold which was subsequently doped with gold and manganese, forming the photocatalyst of formula Au/BiOI/MnO_*x*_; when investigated in the CO_2_ reduction under identical reaction conditions, the CO production rate drastically increased to 42.9 μmol g^−1^ h^−1^, and was accompanied by the formation of trace amounts of CH_4_, O_2_, and H_2_.^106^ Further analysis of the enhanced reactivity observed suggests that using gold and manganese as cocatalyst dopants in this system increase the charge carrier separation efficiency. It was determined that in this process, Au serves as a photo-induced deriving-electron-type cocatalyst (electron acceptor), and Mn operates as a photo-induced deriving-hole-type cocatalyst (hole acceptor). As the conversion of CO_2_ to CO is a reductive process, it would be expected that the gold cocatalyst plays a larger role in the reaction, and indeed, the superior reactivity of Au/BiOI as compared to MnO_*x*_/BiOI supports this theory (*R*_CO_ = 15.3 and 7.21 μmol g^−1^ h^−1^, respectively).

**Fig. 10:**
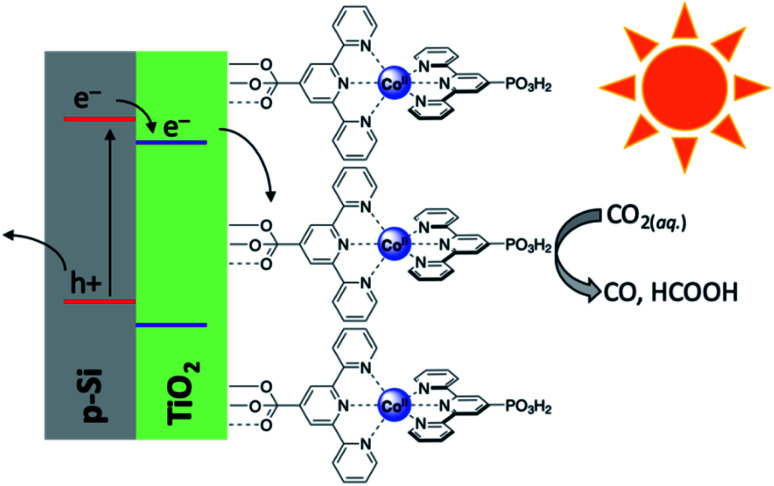
Schematic diagram of p-Si|*meso*-TiO_2_|CotpyP photocathode used in photoelectrolytic reduction of CO_2_.^107^

While the previous study made use of gold only as a dopant, there remains considerable interest in developing a PEC cell which makes use of only earth-abundant metals. It is toward this goal that the Reisner lab has developed an integrated PEC cell driven by a cobalt-based photocathode.^107^ The photocathode was comprised of a p-type silicon electrode, mesoporous TiO_2_ as a scaffold, and an immobilised phosphonated cobalt bis(terpyridine) (CotpyP) catalyst interfaced to the titania layer (Fig. 10). Using the described photocathode in the photoelectrolysis of CO_2_ revealed strong solvent effects on the product distribution and reaction efficiency. Performing this experiment under anhydrous conditions in acetonitrile with tetrabutylammonium tetrafluoroborate (TBABF_4_) as electrolyte showed no catalytic turnover, however under aqueous conditions with KHCO_3_ electrolyte, dihydrogen and formate formed as the dominant reaction products. Systematically varying the H_2_O content in acetonitrile between 10 and 50% with TBABF_4_ altered the product distribution to differing ratios of CO, H_2_, and formate, with the optimum CO production being found at 40% water in acetonitrile. Under these conditions, the turnover number for CO_2_ conversion (TON_CO_2__) was found to be 381 after 24 hours, with *ca.* 75% of gaseous product formed being CO. In additionally analysing the H_2_ and formate formed, it was found that the faradaic efficiency (FE) for CO, H_2_, and formate was 47.6, 16.7, and 12.8%, respectively, and the photocurrent onset potential under the conditions outlined was −0.44 V. Work from the same group soon elaborated upon this work by developing another photocathode composed of a cobalt porphyrin immobilised on buckypaper (CoMTPP@CNT).^108^ Incorporation of this cathode with a BiVO_4_–perovskite photovoltaic device demonstrated the construction of a tandem integrated PEC cell capable of achieving unassisted CO_2_ reduction to CO with simultaneous water oxidation and no detected production of formate. Under 1 sun irradiation, greatly improved turnover numbers for CO and H_2_ were achieved (5853 and 30 725, respectively), and the total faradaic efficiency was calculated to be 91.9%; impressively, even at 0.1 sun irradiation, turnover numbers and efficiencies for this cell significantly outperformed the previously reported CotpyP photocathode (TON_CO_ = 4546, TON_H_2__ = 1359, FE_H_2__ = 17.2%, FE_CO_ = 56.5%, and FE_total_ = 73.7%), illustrating the ability for this cell to operate even under low irradiance conditions.

**Fig. 11:**
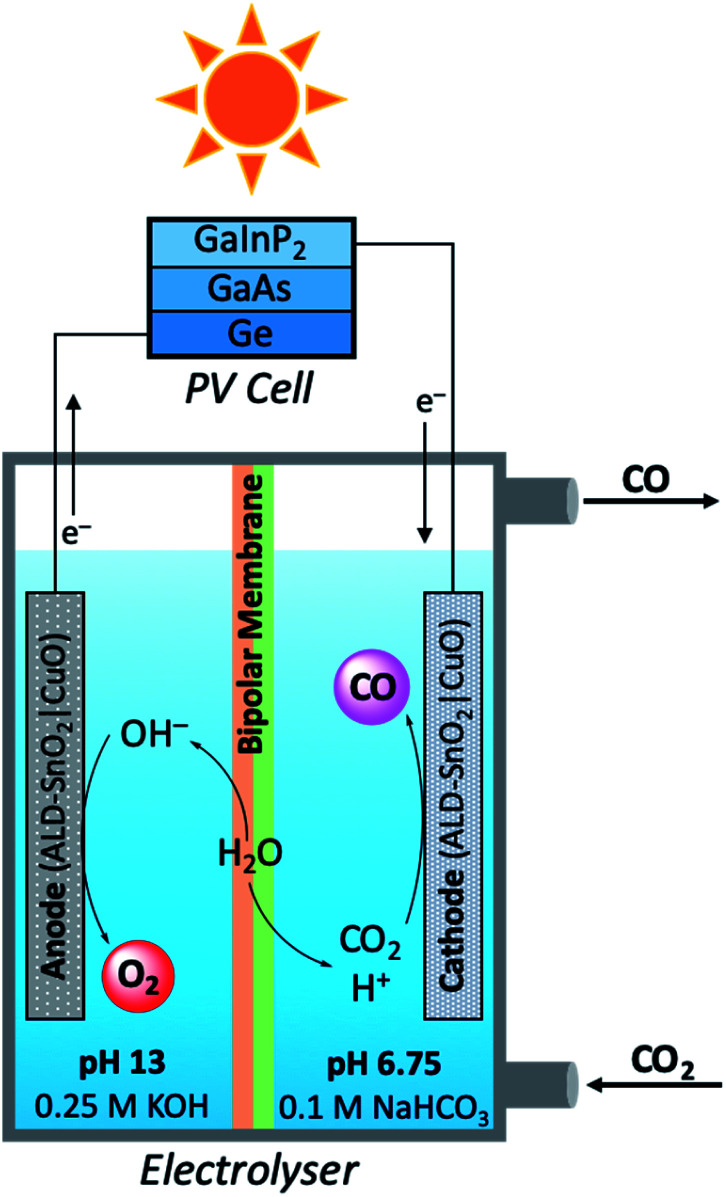
Schematic diagram of modular photoelectrolyser used in carbon dioxide reduction mediated by bifunctional ALD–SnO_2_|CuO electrodes.^109^

In a modular PEC reactor design, Grätzel and co-workers developed a bifunctional electrode composed of CuO nanowires coated with SnO_2_ by atomic layer deposition (ALD).^109^ This work was inspired by previous studies which have shown the utility of CuO as competent electrodes for both electrolytic CO_2_ reduction and water oxidation;^110,111^ a chief difference between the practical aspects of these two reactions is the medium in which the reactions optimally occur. For the aqueous reduction of CO_2_, the oxygen evolving reaction (OER) is most efficient under alkaline conditions, whereas CO_2_ reduction favours pH neutral conditions.^112^ In order to maintain ideal reaction conditions for the oxidation and reduction reaction independently, while minimising the risks of electrode degradation, a PEC reactor was designed consisting ALD–SnO_2_|CuO bifunctional electrodes, a bipolar membrane separating alkaline and neutral reaction media, and a triple junction PV cell (Fig. 11). When the performance of the PEC device was tested in the production of CO at a −0.9 V bias, it was quickly seen that ALD–SnO_2_ modification of the cathode (two ALD cycles) increased the CO turnover frequency drastically to 0.246 s^−1^ as compared to the TOF_CO_ of 0.005 s^−1^ for bare CuO. The product selectivity was also enhanced in this process, evident by the ALD–SnO_2_|CuO TOF_H_2__ being nearly ten times lower than the CuO TOF_H_2__ (0.020 and 0.190 s^−1^, respectively). In addition, the number of SnO_2_ layers deposited on the CuO nanowires proved to be rather consequential; at −0.7 V, maximum CO selectivity was achieved when 2–5 layers of SnO_2_ were deposited by ALD (∼93% CO), and total current density reached its maximum at 5 layers of SnO_2_ with caesium electrolytes (∼1.6 mA cm^−2^). Beyond five ALD layers, the most notable change is the loss in CO selectivity and the increased production of H_2_ and formate. From calculations using the optimised reaction results, the FE_CO_ averaged 81%, the STF_CO_ was equal to 13.4%, and the STF_total_ was calculated to be 14.4%. Perhaps equally as notable as the calculated efficiency values is the observation that the prepared electrodes were not seen to degrade to any observable extent after 5 hours of operation, a problem which frequently plagues PEC reactors.

#### Photoelectrochemical production of liquid solar fuels

The Cat2 production of solar fuels has thus far been limited to gaseous products (CO, H_2_, and mixtures therein), however, the direct synthesis to liquid fuels presents more promising approaches to addressing global fuel needs. Such a transformation proves significantly more challenging though, as CO_2_ reduction to methanol is a 6e^−^ process, whereas the reduction to CO requires only two electrons. Owing to this, reports on photoelectrochemical reduction of CO_2_ to MeOH remain comparatively scarcer; nonetheless, modern research has succeeded in overcoming these barriers to achieve the desired conversion.

**Fig. 12:**
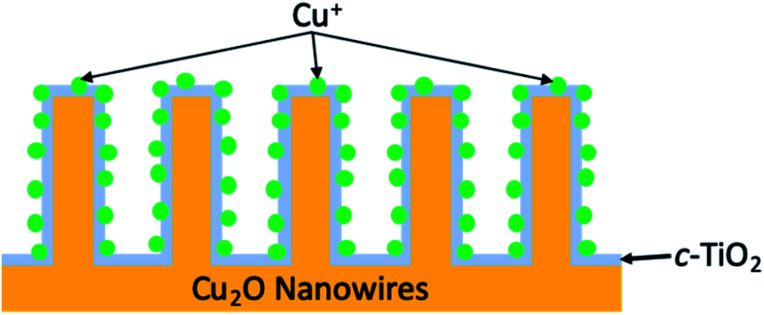
Schematic cross-sectional view of Cu_2_O|TiO_2_–Cu^+^ photocathode.

In 1994, work from the Bocarsly group discovered that under electrochemical conditions, the pyridinium cation was a surprisingly effective catalyst for the reduction of CO_2_ into methanol in the presence of a hydrogenated palladium electrode.^113^ It was theorised that under the described reaction conditions, pyridinium was readily reduced to a pyridinium radical; this intermediate would be expected to provide a source of both protons and electrons, thus allowing for shuttling of these species to effect the production of methanol from CO_2_. Moreover, the low overpotentials (∼200 mV) required to achieve this conversion made this a particularly appealing route for MeOH formation. It was more than a decade later that the same group integrated the electrochemical reaction described to a photovoltaic device.^114^ By incorporation of a p-GaP semiconducting electrode in the presence of a pyridinium catalyst, selective photoelectrochemical reduction of CO_2_ into methanol was achieved with faradaic and quantum efficiencies of nearly 100 and 44%, respectively, at an underpotential of 320 mV relative the thermodynamically predicted half-reaction. Later work by Lee *et al.* developed photocathodes used within an integrated PEC cell to similarly form methanol as a product of the photoreduction of CO_2_.^115^ The photocathodes were constructed of cuprous oxide nanowires (Cu_2_O NW) overcoated with a crystalline titania layer, (creating a p–n junction between Cu_2_O and TiO_2_, respectively), that is further decorated with Cu^+^ ions, abbreviated Cu_2_O|TiO_2_–Cu^+^ (Fig. 12). A CO_2_-saturated 0.3 M KHCO_3_ solution (pH 6.8) served as the reaction medium, and systematic studies of the ideal operating potential showed 0.3 V to be ideal for peak methanol production. The photocathode efficiency of bare Cu_2_O NW was compared to that of Cu_2_O|TiO_2_–Cu^+^ and showed two particular trends in reactivity; (1) bare Cu_2_O NW resulted in remarkably slow reactivity after 30 minutes due to photocorrosion, whereas the novel photocathode provided relatively constant reaction rates, and (2) at any point during the reaction timeline, *R*_MeOH_ of Cu_2_O|TiO_2_–Cu^+^ was at least double that of bare Cu_2_O NW. Observing the faradaic efficiencies and methanol production for both of these systems over the course of a 2 hour reaction revealed that Cu_2_O NW showed a value of 23.6% and generated approximately 0.14 μmol of MeOH, whereas the titania overlayed nanowires more than doubled these values with a FE_MeOH_ of 56.5% and produced ∼0.56 μmol of methanol.

Away from the classical structural motifs of powders, films, and nanostructures being used as scaffolds for photocathodes, Jing and co-workers utilised a nickel foam support to construct a photoactive cathode for methanol production, with a BiVO_4_ film used as the photoanode.^116^ Commercially available nickel foam (f-Ni) sheets served as a support for TiO_2_ which was later functionalised by amine or imine functionality. This was performed using a titania sol–gel followed by annealing to produce TiO_2_|f-Ni, which upon reaction with 3-aminopropyltriethoxysilane (APTES) generated the amine-modified cathode NH_2_–TiO_2_|f-Ni. Subsequent condensation in the presence of salicylaldehyde produced the imine-derived photocathode CHO–NH_2_–TiO_2_|f-Ni, thus providing three different nickel foam electrode motifs which were ultimately studied. When combined in a PEC cell with 0.1 M KHCO_3_, current densities up to −1.1 V were achieved, well beyond the required −0.6 V needed to achieve both water splitting and CO_2_ reduction. In this study, the reactivity of each of the aforementioned nickel foam electrodes was compared, and a screening of the bias voltage applied was performed to optimise reactivity and efficiency. It was found that the methanol production rate for the photocathodes increased in the order of: TiO_2_|f-Ni < NH_2_–TiO_2_|f-Ni ≪ CHO–NH_2_–TiO_2_|f-Ni. Seeing the latter of these photocathodes as the most productive, a screening of the applied bias voltage displayed remarkable results. At −1.0 V, the methanol production rate was 186.5 μM h^−1^ cm^−2^, corresponding to a FE_MeOH_ of 27.3%. If the voltage intensity is decreased to −0.6 V, the *R*_MeOH_ unsurprisingly is lowered to 153.4 μM h^−1^ cm^−2^, however the methanol faradaic efficiency increases drastically to 452.0%, with a FE_total_ equal to 507.9%, and a cell quantum efficiency (*Φ*_cell_) of 1.2%. In addition to methanol product formed in these PEC cells, trace amounts of formic acid, H_2_, CO, O_2_, and even ethanol (EtOH) were detected in the reaction mixtures at each voltage investigated. The significantly improved reactivity seen in CHO–NH_2_–TiO_2_|f-Ni is attributed to two separate factors. First, this photocathode is found to be a much better light harvester as compared to the other nickel foam electrodes, providing a greater impetus to drive the photocatalytic reactions. Second, the presence of the imine functionality serves as a superior scavenger for dissolved CO_2_, thus increasing the degree of CO_2_ adsorption to the photocathode surface.

While the methanol production seen thus far holds considerable promise for the production of useful solar fuels, the ability to directly generate ≥C2 liquid fuels would be even more advantageous owing to the greater energy density contained in such products, *e.g.* the energy density of ethanol *versus* methanol (Table 2). This goal is considerably more challenging, especially when beginning from CO_2_ feedstock, however work such as that presented by the Wang group has presented PEC cells capable of generating ethanol from CO_2_.^117^ Powdered samples of zinc telluride deposited on graphitic carbon nitride (g-C_3_N_4_/ZnTe) were dispersed on indium tin oxide (ITO) to create the photocathode of their PEC cell. When the photocathode described was irradiated while in a CO_2_-saturated 0.1 M KHCO_3_ aqueous solution at a −1.1 V bias potential, ethanol was produced as the major product at a rate of 17.1 μmol cm^−2^ h^−1^ (FE_EtOH_ = 79.3%). Several factors were determined to facilitate this unusual, though highly valuable reactivity. The interface of the ZnTe and g-C_3_N_4_ creates a type-II heterojunction (staggered band gap), improving the charge separation, and thus the efficiency, of the catalysed reaction. The physical contact between the two materials resulting in the formation of a p–n semiconductor junction is further supported as only CO and H_2_ were formed when powders of zinc telluride and carbon nitride were utilised in the PEC cell without any chemical or hydrothermal treatment to create the binary cathode; this theory was also supported by the three fold increase in the standard electron transfer rate of g-C_3_N_4_/ZnTe as compared to ZnTe alone (26.68 × 10^−3^ cm s^−1^ and 7.75 × 10^−3^ cm s^−1^, respectively). Finally, a consideration of the binding affinities of the substrate and intermediates aid in providing a mechanistic explanation to the mode of ethanol production; in comparing the two components of the photocathode, it is found using DFT calculations that CO_2_ has a greater binding affinity to ZnTe, and CO preferentially binds to g-C_3_N_4_, more specifically to the nitrogen atoms which are electronically analogous to pyridine nitrogens. Under the PEC reaction conditions, this leads to a pipeline mechanism wherein, (1) CO_2_ is adsorbed to ZnTe and subsequently reduced to CO on this surface, (2) CO is transferred to g-C_3_N_4_, and (3) photo-induced electron transfer occurs from ZnTe to g-C_3_N_4_, resulting in a high degree of electron density on the conduction band of carbon nitride which drives the C–C coupling and proton-coupled electron transfer to generate ethanol. Of the byproducts formed in the reduction, propyl alcohol was the most abundant (*ca.* 3 μmol cm^−2^ h^−1^) with only trace amounts of CO and H_2_ being observed.

#### Bio-photoelectrochemical hybrid cells for CO_2_ conversion

**Fig. 13:**
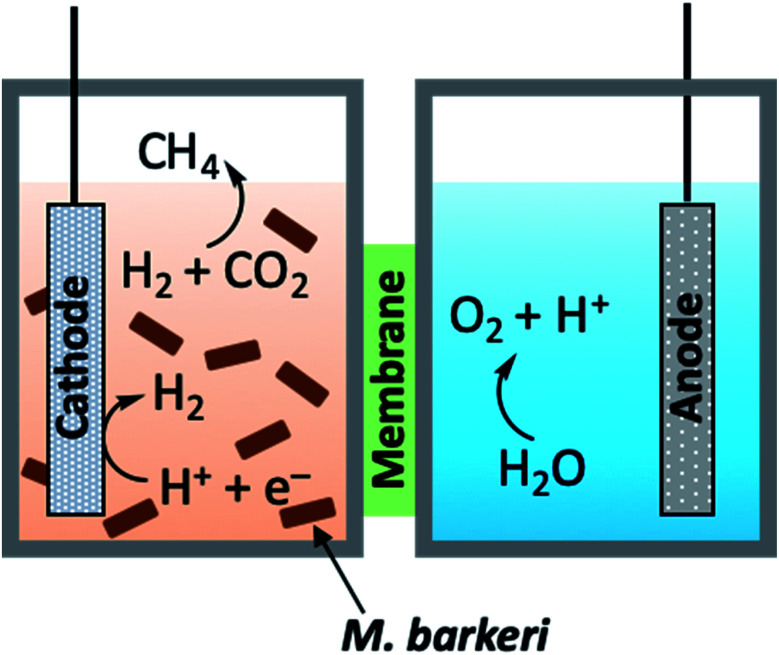
General schematic diagram of hybrid bio-PEC cell.^118^

Autotrophic conversion of CO_2_ into biologically useful energy carriers is a well-established phenomenon which has even been applied to the formation of value-added chemicals (*vide infra*), however, the use of light-harvesting organisms has also found utility in hybrid devices which incorporate man-made technologies to complete the solar-to-fuel cycle. While the delineation between Cat2-bio hybrids and devices falling under Cat4 may be rather tenuous, discussion of the former will be limited to the use of solar transformation which parenthetically includes biological organisms to produce solar fuels. One such example of this is CO_2_ methanation using a bioinorganic hybrid developed by Nichols and co-workers.^118^ In approaching this synthetic challenge, the presented work sought to combine an inorganic-catalysed hydrogen evolving reaction (HER) with biologically-mediated CO_2_ reduction. In developing this device, the more challenging consideration was directed at selecting an organism which would both be compatible with the photoelectrochemical cell conditions, and successfully work in tandem with the inorganic module and the products generated from the reactions therein. This inspired the use of *Methanosarcina barkeri*, an anaerobic prokaryote which is metabolically driven by the 8-proton, 8-electron reduction of CO_2_ to CH_4_.^119^ PEC components were systematically studied in the presence of this archaeon to minimise the electrochemical input needed to achieve the methanogenesis, ultimately leading to the development of an entirely solar-driven hybrid bio-PEC reactor (Fig. 13). As a proof of concept, the reactor was first equipped with a platinum cathode and galvanostatic electrolysis performed at 2.5 mA, resulting in HER at the cathode. From the hydrogen produced in the half-cell, the catholyte containing *M. barkeri* was found to achieve the target CO_2_ methanation, producing CH_4_ gas at a constant rate for up to seven days without any noted decrease in reactivity (109 mL CH_4_ produced in this time). After development and testing of other non-precious metal electrodes, the desired fully solar-driven PEC reactor was created incorporating a nanowire titania (n-TiO_2_) on fluorine-doped tin oxide (FTO) as the photoanode, and platinum-coated p-junction indium phosphide (p-InP) as the photocathode. Owing to previously reported blue-light sensitivity of *M. barkeri*,^120^ a 455 nm filter was interfaced to the reactor, resulting in 1.75 mL of CH_4_ being produced over three days with a farradaic efficiency of 74% for CH_4_ production.

A broader diversity of products was formed by Yang *et al.* through the use of a nanowire-bacteria hybrid PEC reactor and subsequent reaction with engineered bacterial cells.^121^ Using a reactor setup similar to that used in the previous reference, titania (photoanode) and silica (photocathode) nanowire arrays were separated by an ion-conductive membrane with the anaerobic bacterium *Sporomusa ovata* being contained within the catholyte. Under visible light irradiation, the titania semiconductor effected water oxidation to yield protons which traversed the ion membrane; using these protons, electrons produced from the silicon nanowire array, and CO_2_ feedstock, the *S. ovata* converted CO_2_ into acetic acid. If the generated acetic acid was periodically removed from the cell and the lost volume replaced with bacterial growth media, the cell showed indefinite stability up to the maximum 120 hour reaction time. Depending on the culture media used, the hybrid *S. ovata* PEC cell could generate up to six grams acetic acid per litre of electrolyte volume with FE_AcOH_ values up to 70%. Genetically engineered *E. coli* was used to convert the acetate to acetyl coenzyme A (acetyl-CoA), which may then be enzymatically converted to numerous value-added chemicals,^122^ most notable for this review being *n*-butanol. The *n*-butanol was produced up to a concentration of 198 mg per litre of growth medium and with a solar-to-fuel efficiency of 0.20%, however other products were produced from this study including amorphadiene, epi-aristolochene, cadinene, and polyhydroxybutyrate polymer. Though these four products reported are not viable solar fuels, they may still serve as suitable CO_2_-sinks to repurpose greenhouse gas emissions. While the production of acetic acid, acetyl-CoA, and the subsequent enzyme-derived products were all performed separately, it is conceivable that the full process may be streamlined either by possible incorporation into the hybrid PEC device, or by design of a modular reactor to perform these reactions stepwise.

Such a concept as designing a stepwise reaction system was demonstrated from the Schmid research group by incorporating a carbon dioxide photoelectrolyser system with a fermentation module.^123^ A modular photoelectrochemical cell was developed using an external photovoltaic device to create the charge potential needed to power the electrolyser. The cathode used was a commercially available silver-based gas diffusion electrode, and the anode was an iridium-mixed metal oxide (Ir-MMO) coated titania sheet. While the anolyte and catholyte were both aqueous solutions as is commonly employed in PEC reactors, the cell design used was unique in that a gas channel is present behind the cathode to allow direct introduction of CO_2_ into the reactor system without concern for the common limitations of CO_2_ solubility in aqueous media. When irradiated with simulated solar light, the photovoltaic generated 3.65 V which powered the electrolyser and succeeded in reducing CO_2_ to a syngas mixture. It was found that the faradaic efficiency for this process was nearly 100%, and equally as impressive is the consistent reaction rate observed even after running for more than 1200 hours. Owing to the reactor design, very low levels of oxygen flowed with the syngas product (≪100 ppm); the generated syngas was then flowed into a fermenter, and the products of this fermentation were found to be dependent on the bacterial cultures used in this system. If fermentation was performed with *Clostridium autoethanogenum*, acetate and ethanol were formed at a rate of 0.81 and 0.035 mmol h^−1^, respectively. When the bacterial cultures used were *C. autoethanogenum* and *C. kluyveri*, the products of syngas fermentation were acetate, ethanol, butyrate, butanol, hexanoate, and hexanol, each produced at rates of 1.45, 0.6, 0.21, 0.14, 0.05, and 0.04 mmol h^−1^, respectively. This work has also presented an industrial design for implementation of this technology which would potentially produce butanol and hexanol at a scale of 10 000 tonnes per year, illustrating the viability of this solar fuel generation methodology. The researchers additionally pose questions regarding further optimisation of this process by the use of other bacteria or yeast species, perhaps foreshadowing elaboration of this technology toward greater selectivity for higher alcohols and other CO_2_-derived solar fuels.

### Category 3: biotransformation by natural photosynthesis

Photosynthesis is undoubtedly the original solar fuel generating technology with plants representing the prime example, fixing CO_2_ and water to generate glucose as a basic energy source for metabolism. Humanity has long exploited this process as a food source, but additionally as a fuel source to achieve combustion. Primordial biomass combustion was likely performed using wood or other lignocellulosic material, however in the millennia to follow, society has sought to harness cleaner biomass sources as an energy source. By investigation of plants, cyanobacteria, algae, and similar autotrophic organisms, the scientific community has unveiled combustible biofuels created as photosynthetic products from such species; in addition, as the state of the art in bioengineering has improved exponentially over recent decades,^124–126^ our ability to tailor autotrophs toward production of specific photosynthetic products has reached an impressive level of sophistication.^127–133^ In developing the ability to harness natural photosynthesis to generate solar fuels, either using wild-type or genetically modified organisms, several challenges must be mitigated. Some of the most significant of these include blocking photosynthetic pathways which may favour cell growth and biomass production over solar fuel generation, and the selection or engineering of organisms which are not easily poisoned by the fuel generated in a given process. Despite the complexity involved in addressing these concerns, studies of recent years have proved ingenious in their ability to overcome such limitations, especially through the use of cyanobacteria.

A 2012 report demonstrated that a recombinant cyanobacteria (*Synechoscystis* 6803) was capable of generating ethylene by overexpression of the *Pseudomonas* ethylene forming enzyme (EFE), albeit at modest rates.^134^ This work inspired the development of an engineered bacterium (JU547) possessing an enhanced ribosome binding site in the EFE toward forming an ethylene sink in the bacterial metabolism, resulting in significantly enhanced specific ethylene production rates.^135^ The mechanism of ethylene (C_2_H_4_) production was further investigated to determine the factors which improved C_2_H_4_ production in the in JU547 over *Synechocystis* 6803 and other wild-type cyanobacteria; it was found that the citric acid cycle (CAC) in JU547 operates in a closed cycle, whereas this system exists as a bifurcated cycle in wild-type cyanobacteria. The higher efficiency of the engineered bacterium is evident by the greater CO_2_ flux through the CAC, showing 37% total fixed carbon in the closed cycle, a nearly 3-fold increase over the 13% total fixed carbon in the bifurcated system. This was ultimately manifest in the in the ethylene production rate of 718 μL L^−1^ h^−1^ under 730 nm light irradiation.

**Fig. 14:**
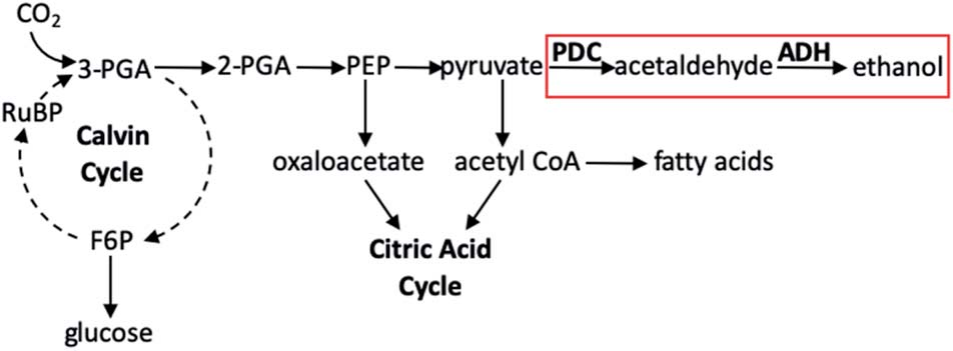
Partial diagram showing photosynthetic steps. Pathway in red box is added pathway from *Synechococcus* sp. strain PCC 7942 genetically modified with *Z. mobilis* PDC and ADH enzymes. Abbreviations: PGA, phosphoglyceric acid; F6P, fructose-6-phosphate; PEP, phosphoenolpyruvic acid; RuBP, ribulose-1,5-biphosphate.^136^

Toward forming liquid fuels with more direct utility, Deng and Coleman integrated the photosynthetic utility of cyanobacteria with the ethanol production of a particular prokaryotic bacterium.^136^ The cyanobacteria used in this study was *Synechococcus* sp. strain PCC 7942, a unicellular organism well known to readily uptake foreign DNA, either through vector shuttling or homologous recombination;^137^ the prokaryote employed was *Zymomonas mobilis*, an especially useful bacterium for this purpose owing to its abundance of the enzymes pyruvate decarboxylase (PDC) and alcohol dehydrogenase II (ADH). In wild-type organisms these genes are necessary for regeneration of NAD^+^ for glycolysis under aerobic conditions in fungi, yeasts, and higher plants, though in organisms which produce high amounts of these enzymes, ethanol production has been seen. Using the *E. coli* plasmid vector pCB4, the PDC and ADH genes of *Z. mobilis* were cloned into this shuttle vector and incorporated into PCC 7942. In preparing the variants of PCC 7942, PDC and ADH gene expression could be controlled by either the cyanobacterial *rbcLS* promoter (the operon encoding for the subunits of ribulose-1,5-bisphosphate carboxylase/oxygenase) or the *E. coli lac* promotoer (the operon required for lactose transport and metabolism). The pCB4-Rpa cell line displayed PDC and ADH expression through the *rbcLS* promoter, whereas the pCB4-LRpa and pCB4-LR(TF)pa cell lines effected gene expression by combinations of control between *rbcLS* and *E. coli lac* promoters. These three cell lines, in addition to a pCB4 control group, were cultured in the presence of light for 21 days at 30 °C, after which cells were harvested to determine PDC and ADH activity from cell lysates, and the culture medium was analysed to determine the ethanol produced by PCC 7942 as a function of final ethanol concentration (Table 3). The highest production activity for the hybrid cyanobacteria was found for pCB4-LR(TF)pa, yielding a final ethanol concentration of 1710 μM (0.23 g L^−1^). Upon further investigation of the mechanism of selective ethanol production, it was determined that the presence of the PDC and ADH genes introduced a new reaction pathway not seen in the unmodified *Synechococcus* sp. PCC 7942 (Fig. 14). The metabolic activity of the cyanobacterium continues to rely on classical photosynthetic steps, including the Calvin cycle; this would generally produce 2-phosphoglyceric acid, which is subsequently transformed into phosphoenolpyruvic acid, then in to pyruvate, both of which may be transformed into other products which fuel the CAC. Rather, in the PDC/ADH-modified PCC 7942, the presence of PDC facilitates transformation of pyruvate into acetaldehyde, and the ADH enzyme converts this to ethanol. Perhaps most notable in this study are the conditions employed to achieve the fermentation to produce ethanol. While many natural photosynthetic processes only produce ethanol under dark, anaerobic conditions, the cyanobacterial–prokaryotic hybrid presented here produced this solar fuel during oxygenic photosynthesis without the need for any other special reaction conditions being applied.

**Table 3:** Ethanol production activity of *Synechococcus* sp. strain PCC 7942 genetically modified with *Z. mobilis* PDC and ADH enzymes^136^

Cell line	Activity^a^ (μmol min^−1^ g of total protein)		Ethanol concentration (μM)
	PDC	ADH	
pCB4 (control)	ND^b^	ND	ND
pCB4-Rpa	130	168	1370
pCB4-LRPa	136	140	1540
pCB4-LR(TF)pa	234	168	1710

a Values reported were the mean of two or three experiments.

b Not detected.

This work was further improved upon in the years to follow using *Synechocystis* sp. PCC 6803 by Dexter and Fu,^138^ as well as Duhring *et al.*,^139^ ultimately showing ethanol production as high as 0.46 and 3.60 g L^−1^, respectively. These studies exploited the endogenous ADH enzyme found in PCC 6803, however a 2012 study by Gao *et al.* successfully combined aspects of these works with the genetic modifications performed on PCC 7942 to produce a recombinant cyanobacterium with even greater ethanol productivity.^140^ A total of nine variants of PCC 6803 were produced in this study, but systematic modification revealed the most active mutant and elucidated the pathway by which the increased ethanol productivity was achieved. In a similar manner to the aforementioned research by Deng and Coleman,^136^ the alcohol producing pathway found in *Z. mobilis* was introduced to PCC 6803 by encoding the genes for PDC, ADH, and the promoter *rbcLS* into the *slr0168* site^141^ of the cyanobacterial genome, producing the Syn-XT43 strain. Comparing the photosynthetic behaviour of this strain to a previously reported Syn-LY2 strain of *Synechocystis*^142^ provided valuable insight to the following iterations of PCC 6803 which were produced. Culturing the cells of both Syn-XT43 and Syn-LY2 under identical conditions in an atmosphere of 5% CO_2_ in air resulted in similar growth rates, however the cell density of the former strain was roughly half of that in Syn-LY2. Given that *Synechocystis* can tolerate ethanol concentrations up to 10.6 g L^−1^ without any notable impacts on cell growth,^138^ ethanol accumulation should have no direct effects on cell growth in this strain. The observed disparity of cell density between these strains was explained by one of two theories: (1) the carbon resources in Syn-XT43 are selectively utilised to produce ethanol rather than biomass, or (2) acetaldehyde accumulation may occur in the medium as a result of reverse catalysis of ADH,^143^ thus resulting in cell toxicity. Measuring the ethanol productivity of Syn-XT43 when sparged with 5% CO_2_ in air showed 0.4 g L^−1^ of EtOH generated, roughly four-fold greater than found when sparging with air alone. The second-generation cyanobacterial hybrid (Syn-ZG25) was formed by overexpressing the endogenous alcohol dehydrogenase gene of *Synechocystis* sp. PCC 6803 (*slr1192*) rather than transplanting the ADH gene of *Z. mobilis*. When cultured under the same conditions as Syn-XT43, the ethanol productivity of Syn-ZG25 was found to be roughly 50% higher (0.6 g L^−1^). Motivated by a previous study which diverted carbon flux toward poly-β-hydroxybutyrate (PHB) production and away from the glycogen pathway,^144^ Gao *et al.* further modified Syn-ZG25 to favour the ethanol-production pathway. The first attempt at this was performed by incorporating the PDC gene from *Z. mobilis* and the endogenous *slr1192* gene into the regions coding for polyhydroxyalkanoate-specific β-ketothiolase (*phaA* or *alr1993*) and polyhydroxyalkanoate-specific acetoacetyl-CoA reductase (*phaB* or *slr1994*), forming the Syn-HZ23 strain of PCC 6803. These modifications were expected to block the PHB synthetic pathway, thus favouring ethanol production; surprisingly, blocking the PHB pathway did not show any appreciation in ethanol productivity as compared to Syn-ZG25. Conversely, when two copies of *Z. mobilis* PDC and endogenous *slr1192* were placed at both the *slr0168* site and the location of the *phaAB* gene, yielding the Syn-HZ24 strain of PCC 6803, the ethanol productivity increased drastically up to 5.50 g L^−1^. This result also served to further support the hypothesis that greater expression of pyruvate decarboxylase and alcohol dehydrogenase in cyanobacteria aids in generating greater amounts of ethanol by directing the carbon flux toward this metabolic pathway. When observing the ethanol production of the Syn-HZ24 mutant under the above described aerobic conditions (5% CO_2_ in air), it was found that the production dropped drastically after 15 days, however, if anoxic conditions were used by sparging with 5% CO_2_ in dinitrogen, ethanol productivity remained relatively constant over the course of 30 days. This finding is especially useful in understanding the parameters necessary for potential future industrialisation of this process using this or other genetically modified organisms.

**Fig. 15:**
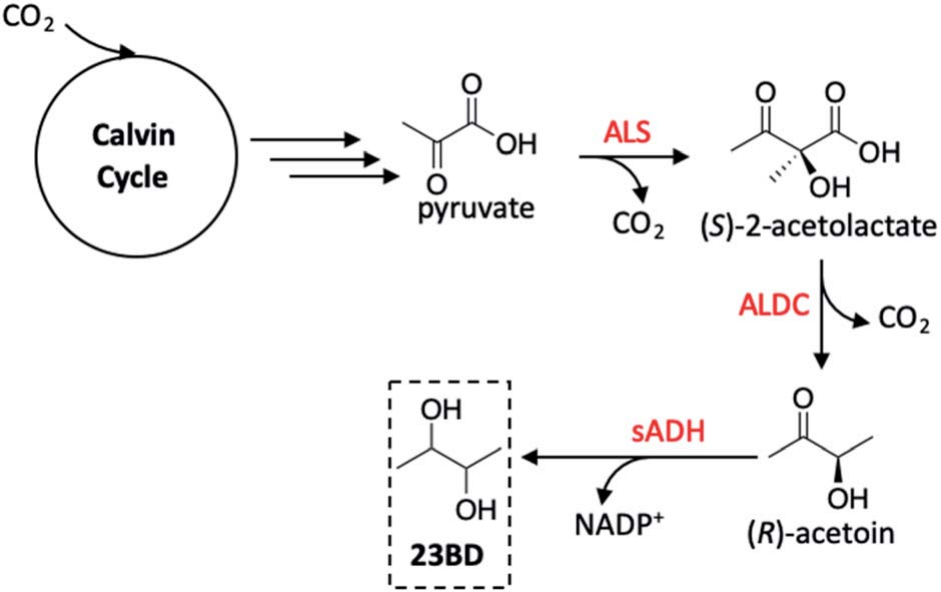
2,3-Butanediol (23BD) production pathway in *Synechococcus elongatus* PCC 7942. Red reagents are enzymes effecting pyruvate to 23BD conversion. sADH may exist as the (*R*) or (*S*) isomer, making the possible products (*R*,*R*), (*meso*), or (*S*,*S*)-23BD. The (*S*,*S*)-isomer is generated by spontaneous conversion of (*S*)-2-acetolactate to diacetyl, ALDC-mediated conversion to (*S*)-acetoin, then reaction with (*S*)-sADH to generate (*S*,*S*)-23BD. Abbreviations: ALS, acetolactate synthase; ALDC, alpha-acetolactate decarboxylase; sADH, secondary alcohol dehydrogenase.^145^

Toward the production of more diverse polyols using GMO's, Atsumi and co-workers utilised *Synechococcus elongatus* PCC 7942 as the host organism as was previously demonstrated by Deng and Coleman.^136^ Rather than targeting ethanol production, however, 2,3-butanediol (23BD) was the product of choice in this study.^145^ Thorough review of precedent literature provided a feasible pathway to achieve this transformation starting from pyruvate (created as a product of the Calvin cycle), generating acetoin as an intermediate product, and ultimately yielding the desired diol (Fig. 15). The first step toward engineering PCC 7942 conversion of CO_2_ into 23BD required constructing a biosynthetic pathway to acetoin production; using *E. coli* as a vector shuttle, *alsS* and *alsD* genes possessing the expressed acetolactate synthase (ALS) were transferred to PCC 7942 to create the *S. elongatus* mutant 2-acetolactate decarboxylase (ALDC) proteins. In all PCC 7942 mutants created in this study, the source of the *alsS* gene was *B. subtilis*, however, *alsD* originated from one of six bacterium, allowing for a systematic investigation of the acetoin production as a function of the gene source. The control strain wherein only *alsS* was expressed produced only 0.2 g L^−1^ acetoin. The low level of acetoin yielded suggests that autodecarboxylation of (*S*)-2-acetolactate is the source of product rather than an enzyme-driven process.^146^ When *alsD* was coexpressed with *alsS*, the acetoin concentration increased up to ten-fold based on the gene carrier used. The highest activity was seen when the *alsD* gene originated from *Aeromonas hydrophila* (21.0 g L^−1^), followed by *Gluconacetobacter xylinus* (17.8 g L^−1^), *Bacillus licheniformis* (16.7 g L^−1^), *Enterobacter aerogenes* (16.0 g L^−1^), and *Bacillus subtilis* (6.6 g L^−1^). It was found that the *Enterobacter cloacae alsD* gene was not active in the *E. coli* vector and was not transferred to PCC 7942, resulting in no discernible increase in acetoin activity. From these results, the PCC 7942 mutant bearing the *B. subtilis alsS* and *A. hydrophila alsD* genes were used in subsequent modifications.

Although acetoin is a valuable chemical to be produced from CO_2_, its toxicity toward *S. elongatus* makes this an unattractive process as this would require continuous removal of product from the culture medium. To generate a more useful product from this system, the incorporation of a secondary alcohol dehydrogenase protein (sADH) was investigated to facilitate the rapid conversion of acetoin to 23BD. In considering the ideal candidates for the gene transfer, additional factors were considered including low oxygen sensitivity, NADPH-dependence (as this characteristic is expected to allow for greater bioavailability during photosynthesis), and the stereospecificity of the enzyme used depending on the desired product. When studying the 23BD productivity to determine the ideal strain for this reaction, it was important to consider both acetoin and 23BD production rates given that slower production of the diol as compared to acetoin would result in a bottleneck in the biosynthetic pathway and toxicity to the bacterial cultures. Four plasmids were tested to create sADH expression in PCC 7942, resulting in the greatest 23BD production in the *alsS* (*B. subtilis*)/*alsD* (*A. hydrophila*)/*adh* (*T. brockii*) strain at a concentration of 952 mg L^−1^ with limited acetoin accumulation (61 mg L^−1^). In long-term studies on the production of 23BD, this strain generated 1.97 g L^−1^ of the target product over three days at an average rate of 7.757 mg L^−1^ h^−1^, however the productivity of the *alsS* (*B. subtilis*)/*alsD* (*A. hydrophila*)/*adh* (*C. beijerinckii*) strain had an even higher 23BD productivity of 2.38 g L^−1^ after three days with an average rate of 9.847 mg L^−1^ h^−1^. This level of 23BD production was sustained for up to 21 days, after which the activity dropped off sharply. Replenishing the culture medium unfortunately did not accelerate 23BD production from the diminished rates, likely arising from spontaneous mutations which revert the cyanobacterial carbon flux toward the natural metabolic pathway of the organism. Though not yet scalable to an industrial process, these findings provide insight to routes of genetic engineering in autotrophs which may aid in improving productivity rates, diversifying products formed to a host of useful solar fuels, and increasing the longevity of the organisms used or created as solar fuel generators.

### Category 4: photoconversion by artificial photosynthesis

Attempts to replicate natural photosynthesis in a man-made, laboratory-scale setting has been a long-standing endeavour of the scientific community. To realise this goal, several attempts have been made to create artificial photosynthesis wherein incident light energy facilitates direct photoconversion of CO_2_ into viable solar fuels.^63,95,147–155^ When considering the general mechanism of chemical reactivity of Cat4 on a quantum level, there are striking similarities to Cat2, especially when comparing to an integrated PEC device. While the following delineation could be qualified as a generality, we will consider such devices which do not require a bias potential or possess a dedicated photoelectrode as Cat4 systems for solar fuel generation. Within this category, researchers have subdivided developed technologies in several ways, *e.g.* suspended photocatalytic powders *versus* photocatalysts deposited on a surface. In this review, Cat4 systems will rather be considered based upon the nature of the catalyst used as either (1) homogeneous catalysts, (2) heterogeneous catalysts, or (3) hybrid bioinorganic catalyst. Each of these subcategories possess inherent advantages and weaknesses which will be discussed in further detail below.

#### Homogeneous catalysts for direct photoconversion of CO_2_

In broadly considering the nature of homogeneous catalysis, several characteristics are commonly understood to be operative in the reaction medium which constrain the bounds of how the reaction proceeds and what information may be obtained from analysis of the mixture and products. Though exceptions do exist, homogeneous reactions are often restricted to liquid-phase reactions; while this is certainly a limitation, the existence of a liquid solution does afford other advantages. Heat transfer and reagent diffusivity is often superior in homogeneous systems as compared to heterogeneous ones, also allowing for lower temperatures to be employed. Moreover, because of the nature in which homogeneous catalysts are prepared and analysed, a high degree of consistency exists between individual molecules within the bulk material, ultimately allowing for a more thorough understanding of the catalyst; this in turn allows for convenient and systematic modification of the catalyst, which may in turn lend itself to a high degree of selectivity. In addition, the array of available analytic techniques to probe a homogeneous solution allows for clear elucidation of the catalyst active site and determination of an operative reaction mechanism. Despite these benefits, there are notable inadequacies in using a homogeneous system, especially downstream in the reaction process. Depending on the target product, separation of the catalyst and isolation of the pure product can prove tedious, often resulting in additional waste being formed, and reduced yields.

**Fig. 16:**
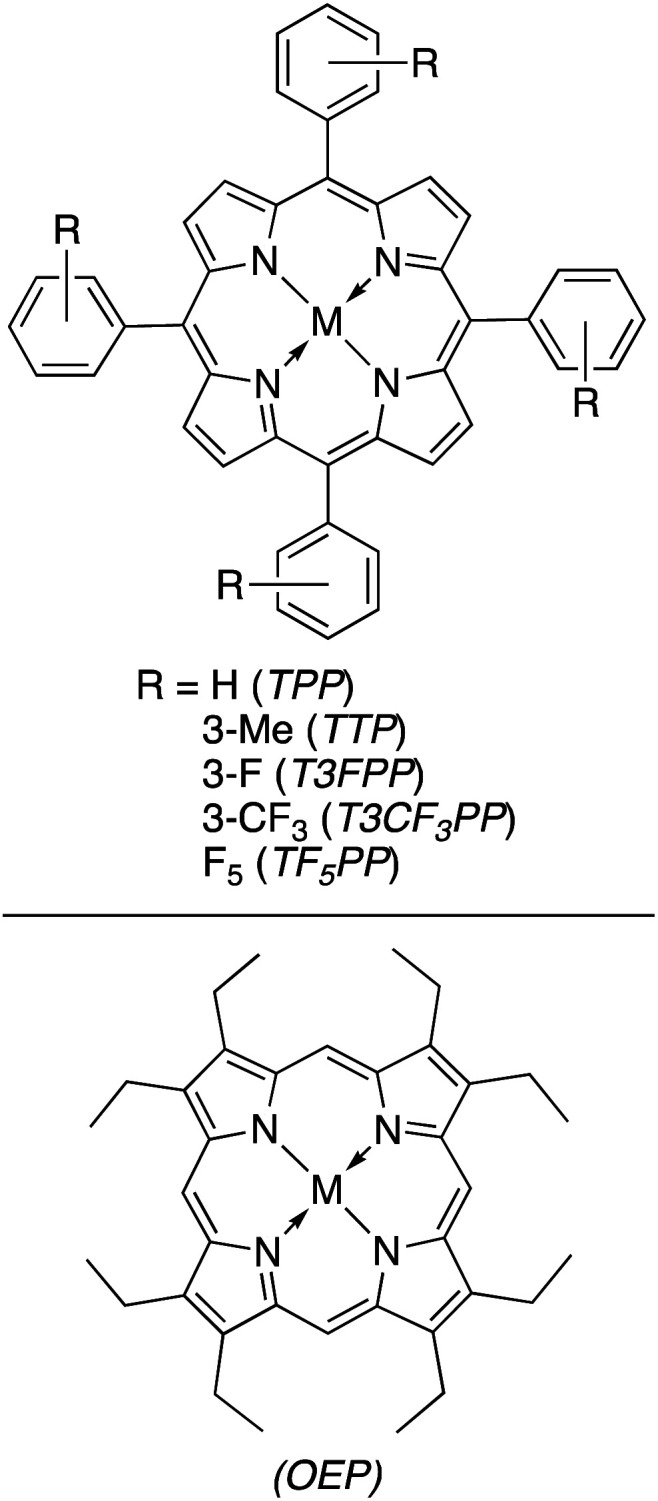
Metalloporphyrins used in the photoreduction of CO_2_.^156^

**Fig. 17:**
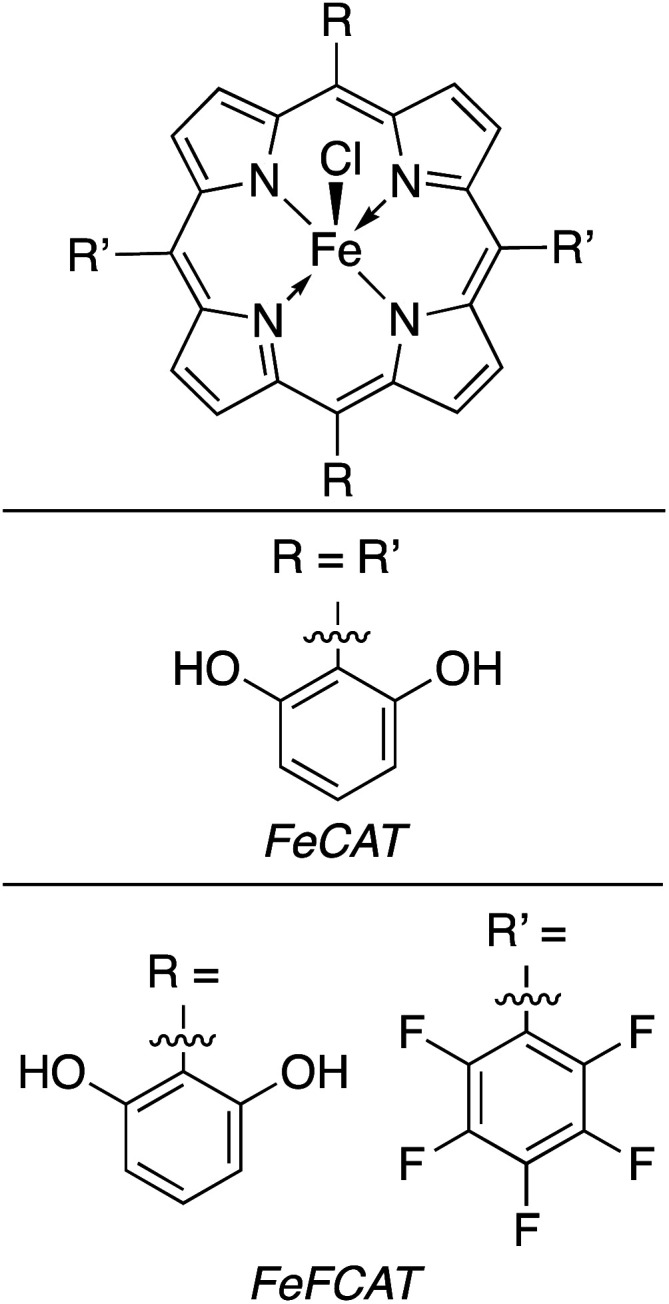
Iron(iii) porphyrin complexes used by Bonin *et al.* in photocatalytic conversion of CO_2_.^157,158^

**Fig. 18:**
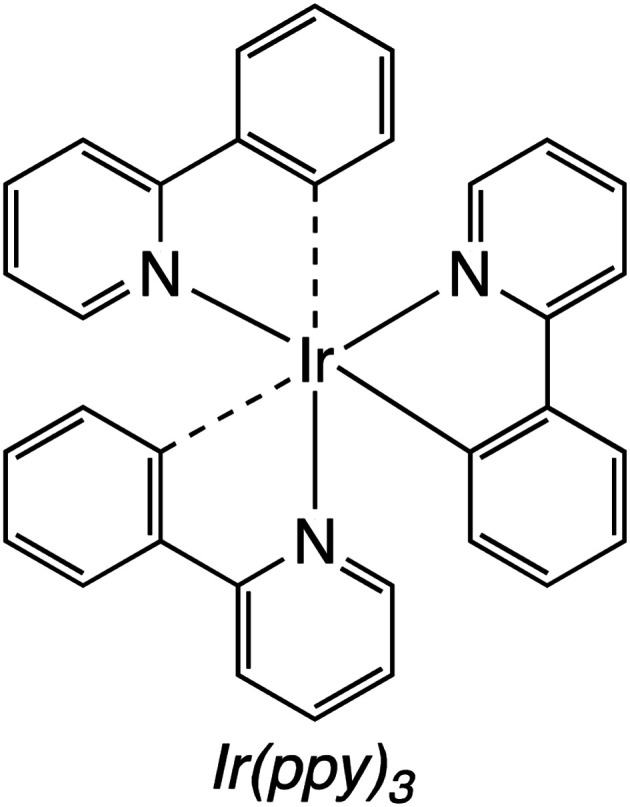
Iridium photosensitiser (Ir(ppy)_3_) studied in the FeCAT-mediated photoconversion of carbon dioxide to carbon monoxide.^158^

**Fig. 19:**
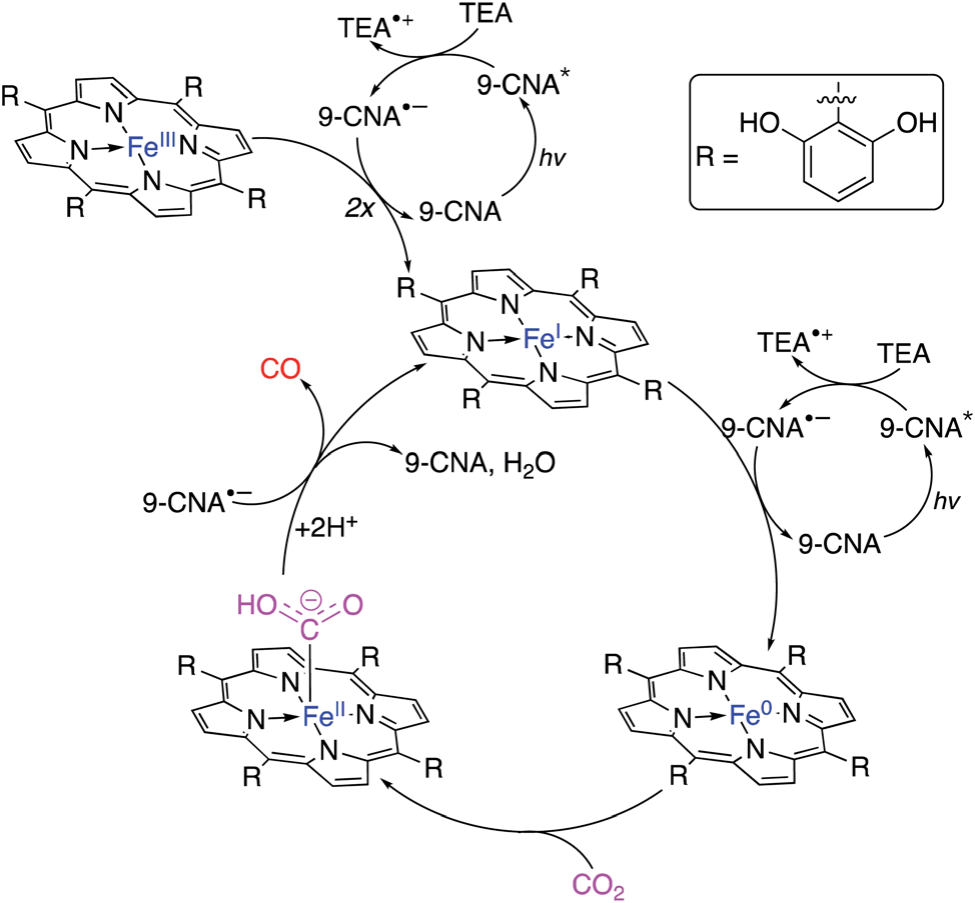
Proposed mechanism of CO_2_ photoconversion to carbon monoxide by FeCAT in the presence of 9-CNA photosensitiser.^158^

Even in relation to solar fuel generation, each of these factors continue to play a role, yet depending on the reaction, the shortcomings discussed can be easily mitigated. In the conversion of CO_2_, gaseous products can easily be separated from the liquid reaction mixtures. Naturally, when the products are liquid fuels, the challenges of separation/purification remain present, however it is most often seen that homogeneous photoconversion of CO_2_ yields either carbon monoxide or syngas mixtures, thus eliminating this challenge. Collaborative work from the labs of Neta and Fujita prepared a series of cobalt and iron porphyrins (MP) toward developing a homogeneous CO_2_ reduction catalyst (Fig. 16).^156^ Screening the activity of the CoTTP and FeTTP metalloporphyrins revealed that maximum conversion occurred in a CO_2_-saturated acetonitrile solution containing 5% triethylamine (TEA) as a reductive quencher, 3 mM *para*-terphenyl (TP) as a sensitizer, and 50 μM of the selected metalloporphyrin. Upon photoactivation, it was found that syngas was formed as the major product of the artificial photosynthesis; the Co^3+^ analogue showed an initial CO and H_2_ production rate of 0.45 and 0.2 mmol L^−1^ h^−1^, respectively, whereas the Fe^3+^ metalloporphyrin generated these gases at an initial rate of 0.84 and 0.1 mmol L^−1^ h^−1^, respectively. After 20 hours of photolysis, the final CO : H_2_ ratios for CoTTP and FeTTP were 3.1 : 1.6 and 2.1 : 3.4, respectively, and it was found that while CO production had generally ceased after this time, H_2_ production from FeTTP continued thereafter. Kinetic and mechanistic studies of this reaction showed that the precatalyst metalloporphyrins are activated by photolytic reduction, yielding M^0^L^2−^ which subsequently reduces CO_2_. The TP introduced to the reaction mixtures serves to increase the quantum yields of MP and CO_2_ reduction by generating a radical anion (TP˙^−^) *via* TEA-mediated reduction. The abovementioned TP radical is found to effectively reduce the MP to its M^0^P^2−^ state. The workers found that accumulation of CO in the reaction medium inhibits further reduction of CO_2_, likely due to CO binding to the metal centre in the MP complex. By screening the dozen MP complexes in this study under these conditions, the highest conversion was found using a 90 μM solution of CoT3FPP, yielding nearly 6 mmol L^−1^ of CO in the final reaction mixture. More than a decade later, Bonin *et al.* similarly showed the utility of metalloporphyrin complexes as homogeneous catalysts for the generation of solar fuels using modified iron(iii) porphyrin complexes (Fig. 17). In a standard experiment, the turnover numbers (TON) and frequencies (TOF) of CO and H_2_ generation were studied as a function of the presence of a weak Brønsted acid, trifluoroethanol (TFE).^157^ The addition of this reagent was attempted to mimic the catalytic activity found for some works which apply a voltage bias. The results from this work showed that the addition of TFE did not generally improve catalytic activity or selectivity (Table 4), however, the iron porphyrins used were found to be comparable to other systems utilising electrochemical reductions of CO_2_ (ref. 159 and 161) without the need for rarer, more costly metal complexes.^162–165^ Unfortunately, the presence of the weak acid resulted in the addition of a catalyst degradation pathway in the proposed mechanism which greatly limited the lifetime of the active catalyst in the cycle. In a further improvement upon this work, the Brønsted acid was removed from the system and an organic photosensitiser, 9-cyanoanthracene (9-CNA), was introduced.^158^ The reaction was performed in CO_2_-saturated solutions of acetonitrile containing 2 μM FeCAT, 0.05 M of TEA, and 0.2 mM 9-CNA. After irradiation with visible light, CO production was consistently achieved over the course of 50 hours with no concomitant formation of H_2_; graphical analysis of the TON for carbon monoxide production revealed a linear plot, indicating that no degradation of the system occurred over the reaction time, a significant improvement over the system using TFE, and either comparable or better than previously reported systems which made use of Ru–Re dyads.^166^ Using 9-CNA as the photosensitiser, a TON_CO_ of 60 was found, and a 100% product selectivity for carbon monoxide was found. Another common photosensitiser, *fac*-(tris-(2-phenylpyridine))iridium(iii) (Fig. 18), was used in this study and ultimately showed superior TON_CO_ of 140, however the product selectivity dropped to 93% in this case. Mechanistic studies of this process allowed for the proposal of a reaction mechanism using 9-CNA (Fig. 19).

TOF and TON for CO and H_2_ formation from CO_2_ mediated by iron(iii) porphyrin complexes^a^

**Table:** 

Catalyst^b^	No TFE			50 mM TFE		
	TOF (h^−1^)		CO selec. (%)	TOF (h^−1^)		CO selec. (%)
	H_2_	CO		H_2_	CO	
FeTPP	5.5	ND^d^	8	5.9	ND^d^	7
FeCAT	0.6	7.7	93	1.2	6.3	85
FeFCAT	2.4	6.7	76	4.3	10.2	73

a CO_2_-saturated solution containing 0.36 M TEA.

b After 1 h of irradiation.

c After 10 h of irradiation.

d Not detected.

**Table:** 

Catalyst^c^	TON (H_2_)		TON (CO)	
	No TFE	50 mM TFE	No TFE	50 mM TFE
FeTPP	37	23	17	7
FeCAT	10	10	28	30
FeFCAT	15	12	23	23

Photoexcitation of 9-CNA followed by reductive quenching by TEA yields the 9-CNA radical anion. Two equivalents of 9-CNA˙^−^ reduce the FeCAT precatalyst to the iron(i) porphyrin active complex; upon reduction with an additional equivalent of 9-CNA˙^−^, Fe^0^CAT is formed which readily binds with CO_2_. Electron transfer from 9-CNA˙^−^ and proton transfer from protonated TEA, both generated *in situ* from the photoactive steps, produces a Fe^II^CAT–CO adduct which, upon additional electron transfer, releases the formed CO with concomitant formation of 9-CNA and water, and regeneration of the active catalyst.

In an attempt to create liquid fuels using homogeneous photoconversion, the MacDonnell research group developed an aqueous catalytic system to convert CO_2_. The catalytic mixture consisted of solvent water, ruthenium(ii) trisphenanthroline (Ru(phen)_3_) as chromophore, pyridine (py) as the CO_2_ reduction catalyst, KCl as electrolyte, and ascorbic acid (AA) as the sacrificial reductant.^167^ The pH of the solution was adjusted using NaOH solution, and blue LED lights (470 ± 20 nm) were used to irradiate samples. The production of methanol and formate under these conditions was systematically investigated by varying concentrations of reagents, and by the addition of metal cocatalysts. Partial optimisation of the reaction showed that a minimum pyridine-to-ruthenium ratio of 100 : 1 is needed to achieve efficient conversion, and the optimum pH for the reaction is approximately 5.0. In addition, the use of potassium salts in the reaction was found to enhance the yield of methanol and formate considerably, however, using other alkali and alkali earth ions had no appreciable affect. After fully optimising this reaction, the ideal conditions were found to be 0.20 mM Ru(phen)_3_, 200 equivalents of pyridine (40 mM), 0.1 M ascorbic acid, and 0.1 M KCl. After adjusting the pH to 5.0 and irradiating with visible light, both methanol and formate were detected in the reaction mixture. After one hour under these conditions, formate was detected as the favoured product with a TON of 76 and a solution concentration of 18 mM; if irradiated for 6 hours, methanol was formed in significantly greater amounts with a TON of 0.33 and a final concentration of 66 μM. Interestingly, beyond six hours, little to no reaction was observed, likely owing to chromophore degradation. In a parallel set of experiments, small amounts of solid metal catalyst was added to the reaction mixture to determine if these would augment methanol production. In all cases (Pt, Pd, Ni and Au on carbon black), the addition of these metals proved inferior to the mixture without the precious metal catalysts, and the additional of colloidal platinum yielded no methanol from the reaction whatsoever.

**Fig. 20:**
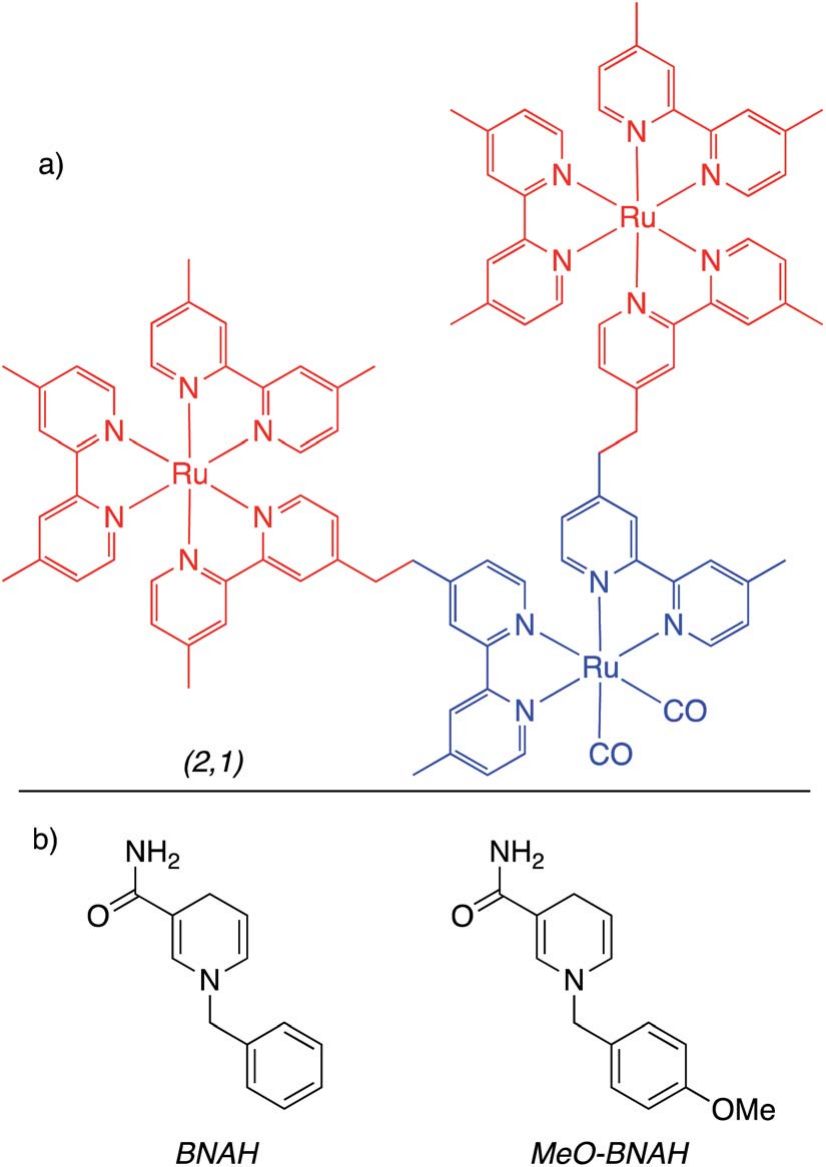
(a) Select supramolecule used in photocatalytic conversion of CO_2_ to formic acid. Red subunits represent photosensitiser, and blue subunit shows catalyst unit. Supramolecular structures in this study are abbreviated according to the photosensitiser-to-catalyst ratio, in this case, (2 : 1). (b) NADH model compounds used as electron donors for the photocatalytic CO_2_ reduction.^168^

In another example of selective formic acid formation from a photoactive homogeneous catalyst, Tamaki *et al.* prepared supramolecular complexes based upon the ruthenium(ii) ion which contained both a photosensitiser subunit and a catalyst subunit.^168^ The photosensitiser base was comprised of Ru^II^(bpy)_3_ units, the catalyst of Ru^II^(bpy)_2_(CO)_2_, and the larger supramolecular structure was constructed by tethering different ratios of these two components together through alkyl bridges which joined adjacent ruthenium(ii) centres by the bipyridine ligands. Using 1-benzyl-1,4-dihydronicotinamide (BNAH) and 1-(4-methoxybenzyl)-1,4-dihydronicotinamide (MeO–BNAH) as NADH model compounds to facilitate electron donation, various conditions were screened to optimise the CO_2_ reduction process. The most productive of these compounds possessed two photosensitiser units to each catalytic unit (2 : 1) and utilised MeO–BNAH as the electron donor (Fig. 20). In a 4 : 1 mixture of *N*,*N*-dimethylformamide/triethanolamine (TEOA), a CO_2_-saturated solution containing a concentration of 0.1 M reductant and 50 μM photocatalyst (2 : 1) was irradiated for 5 hours, yielding 36.8 μmol of formic acid, with small amounts of CO and H_2_ (2.4 and 1.9 μmol, respectively). By comparison, using BNAH as the electron donor produced 30.4, 1.8, and 1.8 μmol of formic acid, carbon monoxide, and dihydrogen, respectively. When the (1 : 2) supramolecular complex was used in the presence of BNAH, the activity dropped drastically, yielding only 8.4 μmol of formic acid with comparable amounts of CO and significantly smaller amounts of H_2_. In more closely observing the (2 : 1)/MeO–BNAH system, the researchers found that for the CO_2_ to formic acid conversion, an impressive turnover number of 671 was found in addition to a TOF of 11.6 min^−1^. It was ultimately theorised that the reaction proceeds by photoexcitation of the photosensitiser unit, followed by reductive quenching of this unit with BNAH, then intramolecular electron transfer from the reduced photosensitiser subunit to the catalyst subunit.

#### Heterogeneous catalytic photoconversion of CO_2_

Just as homogeneous catalyst systems are often characterised by inherent advantages and disadvantages, so too can the general properties of heterogeneous systems be described. Using a heterogeneous catalyst, reactions can be run in the liquid, gas, or solid phase, or even at the interface between two of these phases. As a matter of considerable convenience, the reaction mixture and products can be easily separated from the catalyst at the conclusion of the reaction, and the catalyst can often be recycled or regenerated for reuse in future reactions. However, heterogeneous catalysts frequently suffer from common intrinsic weaknesses. Generally, heat transfer and diffusivity are more problematic in heterogeneous systems owing to the different phases of the reaction medium, and on a molecular level, the active site of the catalyst and reaction mechanism are poorly understood. As a result, heterogeneous catalysts are not easily modified and provide low product selectivity. Several strategies have been developed and utilised within the scope of this review to address and circumvent these challenges toward forming various solar fuels from carbon dioxide feedstock.

**Fig. 21:**
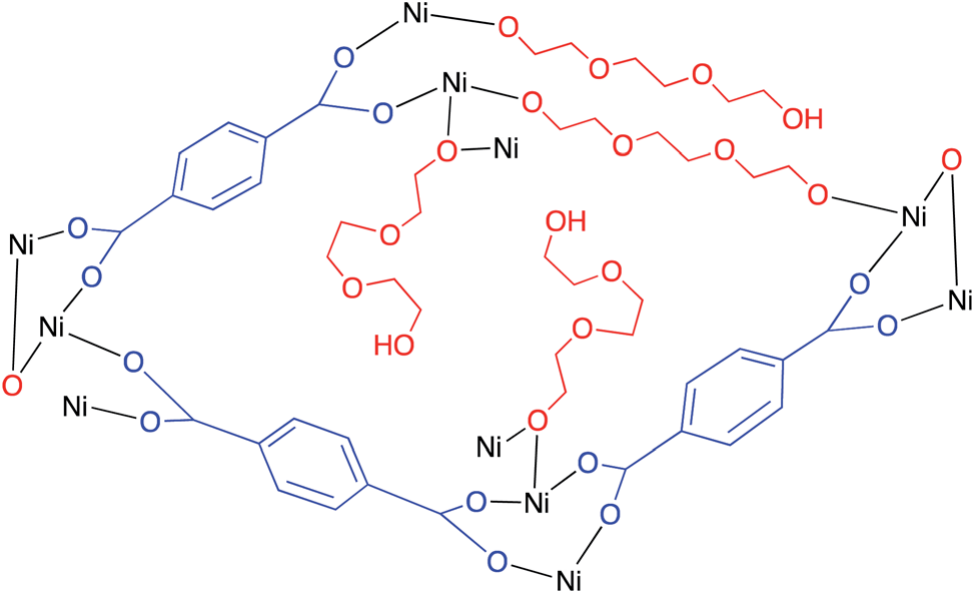
Ni(TPA/TEG) composite formed for the heterogeneous photoconversion of carbon dioxide. Red organic linkers are TEG, and blue organics are TPA.^169^

Niu and co-workers developed a spongy nickel–organic MOF to achieve photocatalytic conversion of CO_2_ into solar fuels using various ligands, metal dopants, and synthetic methodologies.^169^ The organic functionality supporting the MOF structure was terephthalic acid (TPA) and triethylene glycol (TEG), and a series of MOFs were prepared of the generic formula Ni(TPA), Ni(TEG), and Ni(TPA/TEG). In addition, the Ni–organic composites were synthesized using either laser irradiation (*L*) or traditional heating (*H*). These frameworks were prepared by combining solutions of nickel Ni(NO_3_)_2_, TEG, and/or TPA in DMF, stirring for 30 minutes, then heating at 110 °C for 48 hours, or irradiating with a nanosecond pulsed laser for three hours (1064 nm; 10 Hz; 7–8 ns pulse width; 0.9 cm beam diameter; 700 mJ per pulse). Examination of the catalytic activity of these materials revealed that Ni(TPA/TEG) (*L*) displayed the highest activity toward visible-light driven conversion of CO_2_ (Fig. 21). In a standard experiment, 3 mg of catalyst, 2.5 mmol of Ru(bpy)_3_Cl_2_·6H_2_O photosensitiser, and 2 mL of TEOA as sacrificial electron donor were added to an 8 : 2 solution of MeCN/H_2_O. After evacuation of the mixture, the system was pressurised with approximately 53 kPa of CO_2_ and irradiated with a solar simulator. Analysis of the CO_2_ reduction products revealed that carbon monoxide was produced as the major product with no detectable dihydrogen being formed, a remarkable finding as the HER which often accompanies CO_2_ reduction is entirely suppressed in this process. Irradiation for 2 hours generated 95.2 μmol of CO at a rate of 15.866 mmol h^−1^ g^−1^; while kinetic analysis of the evolved CO relative to amount of catalyst showed a linear relationship, suggesting first-order kinetics, decreasing the amount of catalyst to 1 mg of the catalyst under otherwise identical conditions revealed a CO production rate of 26.620 mmol h^−1^ g^−1^. This observation suggests that the relatively greater number of electrons generated from the photosensitiser were more effectively transferred to the catalyst in the reaction medium. Upon isolating and reusing the catalyst in subsequent reactions, the same activity and selectivity was observed, thus demonstrating the considerable durability of the catalyst. In trying to isolate liquid fuels, the nickel–organic composites were decorated with noble metal nanocrystals, namely Rh and Ag (Rh : Ni(TPA/TEG) and Ag : Ni(TPA/TEG), respectively). By comparison, the undecorated Ni(TPA/TEG) produced formic acid and acetic acid at final concentrations of 29.2 and 72.5 μM, respectively; when the noble metal nanocrystals were added, CO production decreased drastically while the liquid fuel generation increased. Rh : Ni(TPA/TEG) formed formic acid as the dominant product at a concentration of 313.5 μM after 2 hours of irradiation, and Ag : Ni(TPA/TEG) generated acetic acid (195.6 μM) as the major product within the same time period.

Using a ZnO-impregnated graphitic carbon nitride (ZnO/g-C_3_N_4_) catalyst, CO_2_ reduction was investigated while varying several factors including: ZnO : g-C_3_N_4_ ratios, reaction time, reaction temperature, and CO_2_ concentration.^170^ Samples containing 2, 4, 6, and 8 weight percent ZnO on g-C_3_N_4_ were prepared and labelled 2ZC, 4ZC, 6ZC, and 8ZC, respectively. Initial investigation of the CO_2_ conversion showed the dominant products of this process to be carbon monoxide, methanol, methane, and ethanol; further optimisation showed 6ZC to be the most active catalyst, which was subsequently used in further studies. In observing variations with reaction time, it was seen that longer reactions were marked by sharp increases in the rate of CO production, while the production rates for the other solar fuels was found to decrease over the course of the reaction. While the effects of temperature were negligible by comparison to other factors stated herein, the CO_2_ reduction rate increased gradually up to 60 °C, then decreased beyond this point. Finally, the CO_2_ concentration as a function of dilution in dinitrogen was investigated and showed that in the presence of water vapor, CO_2_ conversion occurred at a rate of 18 μmol g^−1^ h^−1^, even at a 1% CO_2_ concentration. As the reagent gas concentration rose, the reduction rate was found to continuously increase, even up to 100% CO_2_, thus demonstrating the robustness of the ZnO/g-C_3_N_4_ catalyst. Under these optimised conditions, the CO_2_ reduction was studied for one hour using simulated solar light, showing a reduction rate of 45.6 μmol g^−1^ h^−1^, with a solar fuel production rates for CO, MeOH, CH_4_, and EtOH of 38.7, 19.0, 5.4, and 2.5 μmol g^−1^ h^−1^, respectively. Using a single batch of catalyst for up to six reaction cycles showed a slight decrease in the reduction rate with no notable effect on product selectivity, indicating that the photocatalyst is relatively stable over several runs, but is likely undergoing some level of decomposition. Though not suitable for large scale expansion in its current form, this photoconversion system does display catalytic activity 4.9 and 8.2 times greater than g-C_3_N_4_ alone or P25 titania, respectively, providing guidance for future work to improve upon solar fuel generation from CO_2_.

Although P25 titania was outperformed as a CO_2_ reduction catalyst in the previous study, Sorcar and co-workers prepared and studied a highly active heterogeneous CO_2_ reduction catalyst, beginning from titanium dioxide as the precursor.^171^ P25 was ground with sodium borohydride and annealed under inert atmosphere at 350 °C, yielding reduced blue titania (RBT). Sonication of an RBT dispersion with various amounts of a 2 mg mL^−1^ graphene oxide solution, followed by annealing the recovered solids at 230 °C generated graphene-wrapped RBT (*X*-G/RBT), wherein *X* denotes the volume in millilitres of graphene oxide solution combined with 200 mL of the 10 mg mL^−1^ RBT suspension. Photodeposition of platinum nanoparticles was performed using hexachloroplatinic acid, yielding platinum sensitised samples of the general equation Pt_%_–*X*-G/RBT (% = 0.50, 1.00, 1.25, or 1.50 theoretical wt% platinum). Using the catalyst samples produced, photoreduction experiments were performed under ambient temperature and pressure under continuous gas flow of moist CO_2_, revealing that Pt_1%_–0.50-G/RBT was the most active of the catalysts synthesised. Irradiation of an aqueous suspension of this catalyst under flowing CO_2_ with simulated solar light for seven hours was found to produce a mixture of methane and ethane at rates of 37.0 and 11.0 μmol g^−1^ h^−1^, respectively. The roughly 3 : 1 ratio of methane and ethane produced varied according to the catalyst used, with this ratio being about 2.6 for Pt_1.5%_–0.50-G/RBT, and methane being the only product observed when using pure RBT. The reaction rate was found to decrease significantly after 7 hours, at which point thermo-vacuum treatment at 100 °C for two hours effectively regenerated the catalyst and restored the observed activity; this was theorised to be due to removal of gaseous products which were determined to be likely inhibitors of catalytic CO_2_ turnover, namely ethane. The prepared platinum sensitised graphene-wrapped RBT catalysts also showed excellent long-term stability, showing no loss in activity after 42 hours of operation given vacuum annealing was performed at seven hour increments.

**Fig. 22:**
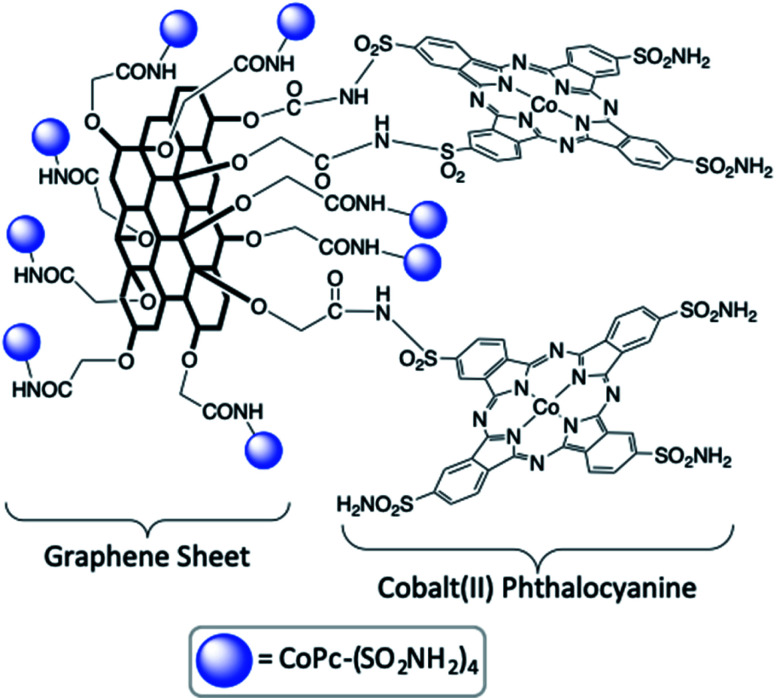
Graphene-tethered cobalt(ii) phthalocyanine catalyst for heterogeneous photoconversion of CO_2_ to methanol.^174^

In addition to gaseous fuels, heterogeneous photoconversion systems have shown excellent utility in generating liquid fuels from CO_2_ as demonstrated by Hsu and co-workers.^172^ In their work, graphene oxide (GO) was prepared by a variety methods to produce materials with different catalytic properties. GO-1 was prepared by an adaptation of Hummer's method by the combination of graphite, sodium nitrate, sulfuric acid, and potassium permanganate.^173^ Addition of different volumes of phosphoric acid followed by standard workup generated the modified graphene oxides, GO-2 and GO-3. The different synthetic methodologies used were found to affect the morphology, and thereby the optical properties, of the isolated material. GO-3 was found to have the roughest surface with the greatest topological variation, as well as the highest bandgap energy of the three materials (3.2–4.4). Consequently, GO-3 was also found to have the highest catalytic activity for photoconversion of CO_2_, generating methanol at a rate of 0.172 μmol g^−1^ h^−1^; while the *R*_MeOH_ in GO-1 and GO-2 increased at comparable rates upon initiation of the reaction up to two hours, the methanol production rate of GO-3 continued to increase up to 4 hours and maintained greater stability over extended reaction times as compared to the other graphene oxide materials. Kumar *et al.* further utilised graphene oxide in the heterogeneous formation of methanol from CO_2_, however the GO used in this study served rather as a solid support for a cobalt(ii) phthalocyanine complex (CoPc–GO, Fig. 22).^174^ Using the catalyst suspended in water with triethylamine as a sacrificial electron donor and 75 W m^−2^ irradiation for 48 hours, methanol was found to be produced as the major product at a rate of 78.79 μmol g^−1^ h^−1^, amounting to 3781.89 μmol MeOH per gram of catalyst. Analysis of the head space of the reaction showed 99.17% of the gas present to be CO_2_ with 0.82% being carbon monoxide as a minor product of the photoconversion. The potential to recycle the CoPc–GO was studied by isolating the catalyst at the conclusion of the 48 hour reaction and determining the identity and weight percent of CoPc remaining on the graphene oxide; after a single run, the cobalt content was diminished by 1.05 wt%, indicating that a small amount of leaching had occurred during the experiment. Though this points to limited recyclability, it also suggests that the heterogeneous catalyst could be used in additional reactions with limited loss of activity.

As the study of heterogeneous photoconverters and PEC devices has shown, seemingly trivial differences in the architecture of the catalyst can result in substantial changes to the chemical behaviour of the system, and research from the Xie lab has demonstrated this concept in their use of bismuth tungstate (Bi_2_WO_6_) layers to achieve conversion of CO_2_ to methanol.^175^ In this work, atomic layers of bismuth tungstate were prepared, and their catalytic activity in solar fuel generation compared to that of bulk and nanocrystalline Bi_2_WO_6_. Hydrothermal treatment of lamellar bismuth oleate with sodium tungstate (Na_2_WO_4_) yielded orthorhombic single-unit cell Bi_2_WO_6_ layers which were expected to display vastly improved CO_2_ conversion efficiencies. The underlying theory of this improved activity was based upon the single-unit cell thickness which would provide a higher specific surface area, an increased density of electronic states, and enhanced charge density on the catalyst surface. Of these factors, the surface area was able to be determined and showed significantly higher values in the atomic layer bismuth tungstate as compared to the bulk Bi_2_WO_6_ (27.6 and 0.7 m^2^ g^−1^, respectively). Performing the photocatalytic reduction using by the atomic layer Bi_2_WO_6_ catalyst with simulated solar radiation for 5 hours yielded 451.7 μmol g^−1^ of methanol as the dominant product, corresponding to an *R*_MeOH_ of 75 μmol g^−1^ h^−1^; this production rate was found to be 3- and 125-times greater than that observed for bismuth tungstate nanocrystals (23 μmol g^−1^ h^−1^) and bulk Bi_2_WO_6_ (0.6 μmol g^−1^ h^−1^). In addition to the accelerated methanol production rate, use of the atomic layer Bi_2_WO_6_ heterogeneous catalyst presented other advantages in the enhanced photostability, demonstrated by the consistent *R*_MeOH_ over the course of six reaction cycles, and the three-fold greater CO_2_ adsorption over bulk bismuth tungstate, likely due to the greater effective surface area discussed above. The activity seen for this bismuth tungstate notably outperformed previously reported atomic layer heterogeneous catalysts, including titania loaded zeolites (5.5 μmol g^−1^ h^−1^),^176^ and Ag/TiO_2_ (4.12 μmol g^−1^ h^−1^),^177^ however a 2017 report from Moriya prepared a titania/zirconia composite which revealed excellent photoconversion of CO_2_ to both methanol and formaldehyde using natural sunlight.^178^ The composite was comprised of nanometre-sized particles of TiO_2_ and micrometre-sized ZrO_2_, and the catalytic and weather conditions meticulously presented showing the impressive catalytic turnover. From combining different ratios of TiO_2_ and ZrO_2_, it was found that 1 : 1 and 6 : 4 ratios of the constituents were the most active in CO_2_ reduction, and mixing and pressing the two oxides produced greater catalytic activity than only mixing them. Water was introduced to the catalyst and resulting mixture by storing samples in a refrigerator to allow condensation to form upon the composite, thereby introducing a thin layer of water atop the oxide. To set a benchmark blank, the photoconversion reaction was attempted using the 1 : 1 composite in the absence of water, and in the dark; performing the reaction without water under solar irradiation showed no turnover of CO_2_, however running with water in the dark showed a low rate of methanol formation over the course of 30 minutes (134 μmol g^−1^ h^−1^), indicating that the catalyst, at minimum, is able to reduce carbon dioxide under classical catalytic conditions. When the wet samples of the 1 : 1 and 6 : 4 composites were irradiated with natural sunlight for five minutes, impressive generation of formaldehyde and methanol was observed, even given the short reaction time. Using the 6 : 4 composite, the sample was refrigerated for 48 hours then exposed to natural sunlight on a clear day (30.4 °C, 67% humidity, 1.16 mW cm^−2^ irradiation intensity); both formaldehyde and methanol were produced at rates of 1392 and 732 μmol g^−1^ h^−1^, respectively. The 1 : 1 composite performed even better under similar conditions (31.2 °C, 68% humidity, 1.33 mW cm^−2^ irradiation intensity), generating formaldehyde at a rate of 3084 μmol g^−1^ h^−1^ and methanol at a rate of 1008 μmol g^−1^ h^−1^. Impressively, even irradiation of the 1 : 1 composite on a cloudy day where the light intensity was 0.45 mW cm^−2^ was found to generate these fuels at notable rates (*R*_HCOOH_ = 312 μmol g^−1^ h^−1^; *R*_MeOH_ = 240 μmol g^−1^ h^−1^); the production of formaldehyde and increased productivity of methanol in this reaction indicate that even under low light, the photoconversion of CO_2_ readily occurs.

#### Hybrid bioinorganic systems for photoconversion of CO_2_

**Fig. 23:**
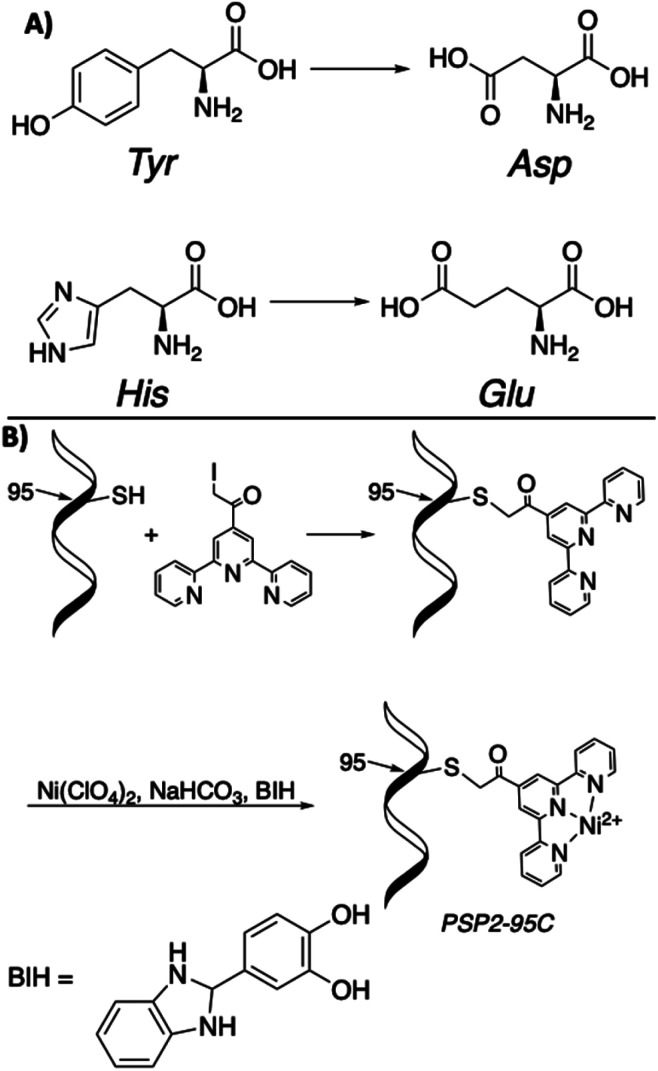
(a) Amino acid residues replaced in sfYFP-BpA66 mutant protein. Tyrosine in 203 position and histidine in 148 position were replaced with aspartic acid and glutamic acid, respectively. (b) Incorporation of terpyridine ligand, nickel(ii) centre, and BIH sacrificial reductant into PSP2 protein, forming PSP2-95C.^179^

As previously discussed in Cat3, attempts to mimic biological processes toward solar fuel production has inspired the use of various organisms to achieve this goal. However, boundaries of the synthetic utility of naturally occurring organism, as well as the challenges associated with creating GMO's capable of producing desirable solar fuels, has limited their use in targeted photoreduction of CO_2_. This has not limited the incorporation of such organisms or enzymes in laboratory-prepared devices toward direct photoconversion. Such hybrid photocatalysts present even greater challenges as the environment must be capable of supporting the chemical and biological components while still facilitating effective CO_2_ reduction. Though limited in number, especially by comparison to the solar fuel generators discussed thus far, successful attempts at creating such a system have been demonstrated and show excellent conversion efficiencies. One such example wherein CO_2_ is photoconverted to carbon monoxide was presented in a 2018 study by Lie *et al.*^179^ Within this work, a genetically encoded photosensitiser protein (PSP) was modified to allow for site-specific coordination of a transition metal complex to serve as the CO_2_ reduction catalyst. In designing the target photoharvesting protein, several initial considerations had to be met, including efficient visible light absorption, a long-lived excited state which would facilitate electron transfer (a characteristic not often seen in proteins), and the ability for the generated excited state to serve as a strong reducing agent. To create a tailored PSP, the superfolder yellow fluorescent protein (sfYFP) was utilized as the scaffold for further engineering. A series of modifications were studied to identify the genetic expansion needed to create a successful CO_2_ reduction system, including variation of the genetic sequence of sfYFP, the sacrificial reductant, and the catalyst. The fluorescent protein was first transformed into a photosensitiser protein by replacing the Tyr66 residue with benzophenone–alanine, forming the sfYFP-BpA66 mutant. The benzophenone is utilised owing to its excited state lifetime which is known to be up to 5 orders of magnitude greater and favours sacrificial reduction.^180–182^ To prevent charge recombination facilitated by π-stacking interactions of proximal amino acids, the Tyr203 and His 148 residues were substituted with aspartate and glutamate, respectively, yielding the mutant protein sfYFP-BpA66–Asp203Glu148 (PSP2, Fig. 23a). From PSP2, select cysteine mutations were added to specific sites of the protein which were used to covalently attach a terpyridine ligand; from these variants, nickel perchlorate (Ni(ClO_4_)_2_) and sodium bicarbonate (NaHCO_3_) were added in the presence of 4-(2,3-dihydro-1*H*-benzo[*d*]imidazole-2-yl)benzene-1,2-diol (BIH) as a sacrificial reductant, quantitatively forming the bioinorganic hybrid photoreduction catalyst (Fig. 23b). The various cysteine sites were selected in order to determine the ideal catalyst-chromophore (*D*_C–C_) distance necessary to favour reduction of the nickel–terpyridine complex over charge recombination. Using crystallographic analysis, the researchers determined that of the five different cysteine mutation sites, the *D*_C–C_ value ranged from 6.0 to 21.9 Å. Applying each of these hybrids in the photoconversion of CO_2_ revealed that substitution at the 95 position (PSP2-95C, *D*_C–C_ = 11.9 Å) showed the best turnover of CO_2_ to CO (TON_*t*=24h_ = 97). While PSP2-95C possessed the most promising photophysical properties of the hybrids generated in this study, the photoconversion was further improved by the incorporation of proton donors near the catalyst site to facilitate photon-coupled electron transfer to the carbon dioxide substrate. This was accomplished by the introduction of two tyrosine residues near the covalent linker of the organometallic complex, specifically at the 93 and 97 positions of the protein, yielding PSP2-95C93Y97Y (PSP2T2). Under similar reaction conditions, the turnover number for the PSP2T2 bioinorganic hybrid was seen to improve nearly 24%, resulting in 120 catalytic turnovers of CO_2_ to CO in the same 24 hour period.

**Fig. 24:**
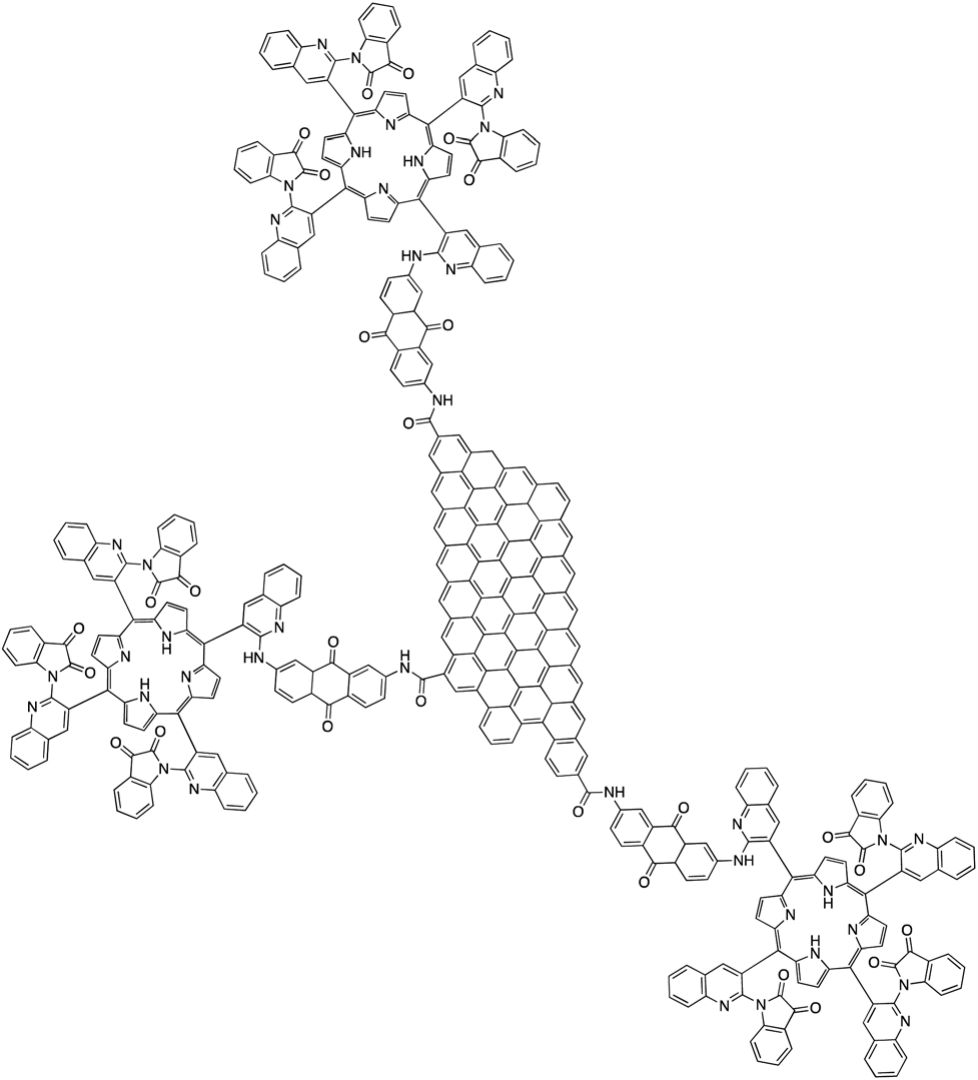
CCG-IP photocatalyst used in photocatalytic/biocatalytic reduction of CO_2_ to methanol.^183^

**Fig. 25:**
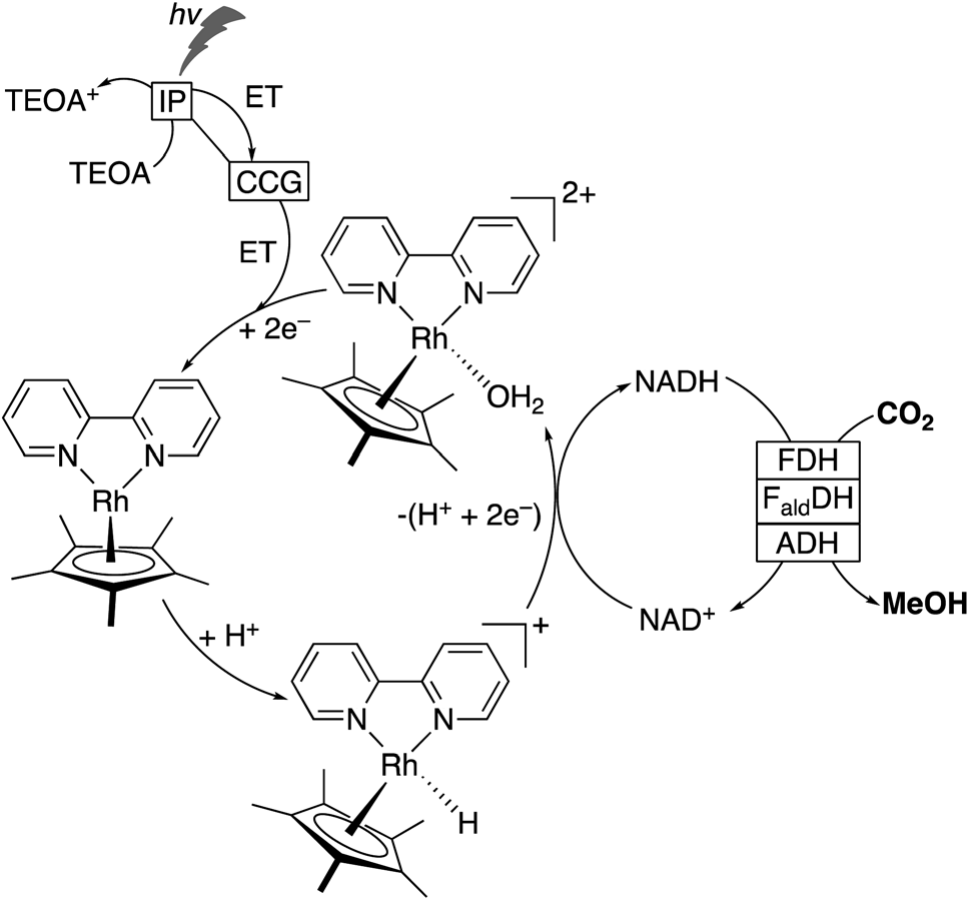
Proposed mechanism of photocatalytic/biocatalytic conversion of carbon dioxide to methanol.^183^

In another work by Yadav *et al.*, individual components of a larger CO_2_ reduction biocatalyst system were replaced with chemical derivatives rather than genetically engineering specific enzymes or proteins.^183^ The basis of this work revolved around the formate dehydrogenase (FDH)–formaldehyde dehydrogenase (FaldDH)–alcohol dehydrogenase (ADH) enzyme cascade which reduces CO_2_ to methanol. Electrons and protons are supplied to the enzymatic reaction *via* the NADH–NAD^+^ cycle, however in nature, stoichiometric amounts of NADH cofactors are necessary to regenerate NADH and support enzyme-mediated CO_2_ conversion. To avoid the use of such cofactors, a tandem photocatalyst cycle, inspired by previous studies on electrochemical regeneration of NADH,^184,185^ was developed to create an alternative catalytic cycle for methanol production from CO_2_ feedstocks. The proposed hybrid photocatalyst/biocatalyst (PC/BC) system requires an efficient light harvester capable of generating electrons and protons which are subsequently shuttled to NAD^+^, thus forming NADH. The researchers previously reported a chemically converted graphene coupled multi-anthraquinone-substituted porphyrin photocatalyst (CCGCMAQSP) which displayed photochemical conversion of CO_2_ to formic acid,^186^ however when used to generate methanol under the hybrid PC/BC conditions, only 5.62 μmol of methanol were formed after 90 minutes of irradiation. Testing chemically converted graphene (CCG) provided even poorer results, yielding only trace amounts of methanol after this reaction time. The results pointed to the need for porphyrin-based light harvesters to be covalently bound to the CCG, similar to what was seen in the CCGCMAQSP photocatalyst, albeit with substituents other than the anthraquinone previously used. To this end, isatin substitution on the porphyrin backbone was used, forming 1,1′,1′′-((20-(2-((7-amino-9,10-dioxo-9,10-dihydroanthracen-2-yl)amino)quinoline-3-yl)porphyrin-5,10,15-triyl) tris (quinolinne-3,2-diyl)) tris(indoline-2,3-dione), referred to as IP. Covalently linking IP to the chemically converted graphene formed the CCG-IP photocatalyst, an efficient visible light harvester (Fig. 24) which proved adept for the desired transformation. The CCG-IP photocatalyst and the FDH–FaldDH–ADH enzymatic biocatalyst served as the basis for the photoconversion, and triethanolamine (TEOA) and Cp*Rh^III^(bpy) were introduced as a sacrificial reductant and electron transfer shuttle, respectively. From electrochemical and mechanistic investigation, it was determined that photoexcitation of IP forms an excited state which transfers electrons to CCG, forming the graphene radical (CCG˙). The resulting IP^+^ is reduced by TEOA, forming the neutral IP and TEOA^+^. The rhodium catalyst abstracts a proton from solution, forming Cp*Rh^III^(bpy)(H) which then regenerates NADH from NAD^+^ by the photo-induced transfer of two electrons and a proton, with concomitant formation of Cp*Rh^III^(bpy)(OH_2_). The rhodium–aqua complex is reduced to its active form (Cp*Rh^I^(bpy)) by CCG˙, and the regenerated NADH is used in the enzyme-mediated conversion of CO_2_ (Fig. 25). Bubbling CO_2_ at a rate of 0.5 mL min^−1^ through the aqueous reaction solution for 60 minutes under visible light irradiation resulted in the formation of 11.21 μmol of methanol (*R*_MeOH_ = 14.95 mmol·g_CCG-IP_^−1^ h^−1^). From the experimental results, it was evident that isatin was superior as a photoabsorber and charge transfer reagent for the electron transfer to CCG as compared to CCGCMAQSP. In addition, the regeneration of NADH, which was one of the chief challenges of this reaction, was more efficiently accomplished by CCG-IP than CCGCMAQSP (38.99 and 28.46%, respectively). This study represents the first example of exclusive methanol formation from CO_2_ by a PC/BC hybrid system and informs of valuable methodologies by which bioinorganic photocatalysts may be developed to create solar fuels from carbon dioxide.

## Outlook

Global climate change caused largely by CO_2_ emissions remains the most pressing environmental crisis facing society today. This threat in combination with the limited supply of fossil fuels prompts the necessity to find alternative energy sources to mitigate CO_2_ emissions and identify sustainable energy sources for long term use. To address both of these concerns, strategies of converting CO_2_ into viable solar fuels have emerged as promising, carbon–neutral options to sustainably generate energy carriers. Research in recent decades has resulted in impressive works which have created innovative routes to generate gas and liquid fuels from a CO_2_ feedstock, yet despite the progress made in this field, many challenges remain which must be overcome before a global implementation strategy can be pursued.

Perhaps most pressing is the improvements needed in the technologies presented, including determination of the long-term stability of systems and further improvement/optimisation of solar fuel production. When aiming to store solar energy in chemical bonds as is proposed, the intermittent availability of sunlight limits the ability to harvest incident solar energy; in order to improve solar-to-fuel outputs, large areas are often required which incidentally creates other challenges in terms of land availability. In most instances, large scale pilot plants which have been constructed are located in isolated areas, thereby adding considerable transportation costs to the expenditures needed for wide-spread distribution. In order to avoid this and decrease the cost per unit of fuel, the efficiency and selectivity of solar fuel generation must be improved. Fortunately, as additional resources are dedicated to this work, it is increasingly likely that new technologies will be developed to achieve CO_2_ photoreduction to solar fuels on an industrial scale.

A further challenge to the implementation of solar fuel technology is the utilisation of CO_2_. In the vast majority of research dedicated to this process, concentrations of CO_2_ well beyond those found in the atmosphere are required to efficiently generate gaseous or liquid fuels. Alternatively, two general approaches have been investigated to maximise CO_2_ utilisation. The first includes direct use of CO_2_ in atmospheric or marine environments to create fuels,^187^ but the more widely investigated is direct carbon capture from power plants.^188–190^ The latter of these is the more promising design, providing a closed system wherein the combustion of solar fuels for energy production generates CO_2_ which is photoreduced to regenerate the solar fuel.

Several opportunities exist to utilise waste CO_2_ to produce energy carriers as viable replacements for conventional fossil fuels. Though several other alternative energy sources exist such as wind, hydropower, and others, harnessing solar energy in the form of chemical bonds in hydrocarbon fuels presents the only option which may conveniently be integrated into the current existing infrastructure. To realise this goal, additional resources are needed toward the fundamental research of creating new solar fuel technologies, as well as in capital investments which allow for expansion and industrialisation of discovered processes.

## Conflicts of interest

The authors declare no competing interests.

## Supplementary Material
